# Ancient, independent evolution and distinct molecular features of the novel human T-lymphotropic virus type 4

**DOI:** 10.1186/1742-4690-6-9

**Published:** 2009-02-02

**Authors:** William M Switzer, Marco Salemi, Shoukat H Qari, Hongwei Jia, Rebecca R Gray, Aris Katzourakis, Susan J Marriott, Kendle N Pryor, Nathan D Wolfe, Donald S Burke, Thomas M Folks, Walid Heneine

**Affiliations:** 1 Laboratory Branch, Division of HIV/AIDS Prevention, National Center for HIV/AIDS, Viral Hepatitis, STD, and TB Prevention, Centers for Disease Control and Prevention, Atlanta, GA 30333, USA; 2 Department of Pathology, Immunology and Laboratory Medicine, College of Medicine, University of Florida, Gainesville, FL 32610, USA; 3 Department of Zoology, University of Oxford, Oxford, OX1 3PS, UK; 4 Department of Molecular Virology & Microbiology, Baylor College of Medicine, Houston, Texas 77030, USA; 5 Stanford University, Program in Human Biology, Stanford, CA 94305, USA; 6 Global Viral Forecasting Initiative, San Francisco, CA 94105, USA; 7 Graduate School of Public Health, University of Pittsburgh, Pittsburgh, PA 15261, USA; 8 Southwest National Primate Research Center, San Antonio, TX 78227, USA

## Abstract

**Background:**

Human T-lymphotropic virus type 4 (HTLV-4) is a new deltaretrovirus recently identified in a primate hunter in Cameroon. Limited sequence analysis previously showed that HTLV-4 may be distinct from HTLV-1, HTLV-2, and HTLV-3, and their simian counterparts, STLV-1, STLV-2, and STLV-3, respectively. Analysis of full-length genomes can provide basic information on the evolutionary history and replication and pathogenic potential of new viruses.

**Results:**

We report here the first complete HTLV-4 sequence obtained by PCR-based genome walking using uncultured peripheral blood lymphocyte DNA from an HTLV-4-infected person. The HTLV-4(1863LE) genome is 8791-bp long and is equidistant from HTLV-1, HTLV-2, and HTLV-3 sharing only 62–71% nucleotide identity. HTLV-4 has a prototypic genomic structure with all enzymatic, regulatory, and structural proteins preserved. Like STLV-2, STLV-3, and HTLV-3, HTLV-4 is missing a third 21-bp transcription element found in the long terminal repeats of HTLV-1 and HTLV-2 but instead contains unique c-Myb and pre B-cell leukemic transcription factor binding sites. Like HTLV-2, the PDZ motif important for cellular signal transduction and transformation in HTLV-1 and HTLV-3 is missing in the C-terminus of the HTLV-4 Tax protein. A basic leucine zipper (b-ZIP) region located in the antisense strand of HTLV-1 and believed to play a role in viral replication and oncogenesis, was also found in the complementary strand of HTLV-4. Detailed phylogenetic analysis shows that HTLV-4 is clearly a monophyletic viral group. Dating using a relaxed molecular clock inferred that the most recent common ancestor of HTLV-4 and HTLV-2/STLV-2 occurred 49,800 to 378,000 years ago making this the oldest known PTLV lineage. Interestingly, this period coincides with the emergence of *Homo sapiens sapiens *during the Middle Pleistocene suggesting that early humans may have been susceptible hosts for the ancestral HTLV-4.

**Conclusion:**

The inferred ancient origin of HTLV-4 coinciding with the appearance of *Homo sapiens*, the propensity of STLVs to cross-species into humans, the fact that HTLV-1 and -2 spread globally following migrations of ancient populations, all suggest that HTLV-4 may be prevalent. Expanded surveillance and clinical studies are needed to better define the epidemiology and public health importance of HTLV-4 infection.

## Background

Deltaretroviruses are a diverse group of human and simian T-lymphotropic viruses (HTLV and STLV, respectively) that until lately were composed of only two distinct human groups called HTLV types 1 and 2 [1-7]. Two new HTLVs, HTLV-3 and HTLV-4, were recently identified in primate hunters in Cameroon effectively doubling the genetic diversity of deltaretroviruses in humans [6,8]. Collectively, members of the HTLV groups and their STLV analogues are called primate T-lymphotropic viruses (PTLV) with PTLV-1, PTLV-2, and PTLV-3 being composed of HTLV-1/STLV-1, HTLV-2/STLV-2, and HTLV-3/STLV-3, respectively. The PTLV-4 group currently has only one member, HTLV-4, since a simian counterpart has yet to be identified [6].

STLV-1 has a broad geographic distribution in nonhuman primates (NHPs) in both Asia and Africa thus providing humans with historical and contemporaneous opportunities for exposure to this virus [2,4,5,9,10]. Indeed, phylogenetic analysis of simian T-lymphotropic viruses type 1 (STLV-1) and global HTLV-1 sequences suggests that different STLV-1s were introduced into humans multiple times in the past resulting in at least six phylogenetically distinct HTLV-1 subtypes [1-5,11]. Recently, a new HTLV-1 subtype was found in Cameroon that was closest phylogenetically to STLV-1 from monkeys hunted in this region and which shared greater that 99% nucleotide identity [6]. Since similar high sequence identities are typically seen in both vertical and horizontal linked transmission cases of HTLV-1 [12-14], the finding of this new HTLV-1 subtype in Cameroon suggests a relatively recent cross-species transmission of STLV-1 to this primate hunter and that these zoonotic infections continue to occur in persons naturally exposed to NHPs.

Although a simian T-lymphotropic virus type 2 (STLV-2) has been identified in two troops of captive bonobos (*Pan paniscus*), the zoonotic relationship of this divergent virus to HTLV-2 is less clear [15-17]. Like STLV-1, STLV-3 also has a broad and ancient geographic distribution across Africa [9,10,18-23]. Thus, while only three distinct HTLV-3 strains have been identified to date in Cameroon [6,8,24], it is conceivable that HTLV-3 may be prevalent throughout Africa and, like HTLV-1 and HTLV-2, potentially could be spread globally through migrations of infected human populations. Expanded screening is needed to define the prevalence of HTLV-3 in human populations. Likewise, the epidemiology of HTLV-4 is not well understood since only a single human infection has been reported and a simian counterpart has yet to be identified [6]. Although limited sequencing of very small gene regions showed that HTLV-4 is most genetically related to STLV-2 and HTLV-2, but is a distinct lineage separate from all known PTLVs [6], understanding the evolutionary relationship of HTLV-4 to known PTLVs requires additional phylogenetic analyses using longer sequences or the complete viral genome.

Like HIV, both HTLV-1 and -2 have spread globally and are pathogenic human viruses [1,2,5,7,25]. HTLV-1 causes adult T-cell leukemia/lymphoma (ATL), HTLV-1 associated myelopathy/tropical spastic paraperesis (HAM/TSP), and other inflammatory diseases in less than 5% of those infected [2,5,7]. HTLV-2 is less pathogenic than HTLV-1 and has been associated with a neurologic disease similar to HAM/TSP [1]. The recent identification of HTLV-3 and HTLV-4 in only four persons limits an evaluation of the disease potential and secondary transmissibility of these novel viruses [6,8,24]. However, complete genomic sequences of these viruses can provide insights on the genetic structure and whether functional motifs that are important for viral expression and HTLV-induced leukemogenesis are preserved [6,8,24,26-30]. In addition, determination of the viral sequence will be important to develop improved diagnostic assays to better understand the epidemiology of this novel human virus.

In this paper, we report the first full-length sequence of HTLV-4 and demonstrate by detailed phylogenetic analysis that this virus clearly falls outside the diversity of all other PTLVs. The observed low nucleotide substitution rate, absence of evident genetic recombination, and conserved genomic structure of HTLV-4 demonstrate the genetic stability of this virus. In addition, molecular dating suggests that the HTLV-4 lineage split from the progenitor of PTLV-2 about 200 millennia ago and is older than the ancestors of HTLV-1, HTLV-2, and HTLV-3. We also highlight biologically important molecular features in HTLV-4 that are unique or common to HTLV-1, HTLV-2, and HTLV-3.

## Results

### Comparison of the HTLV-4(1863LE) proviral genome with prototypical PTLVs

**Figure 1:**
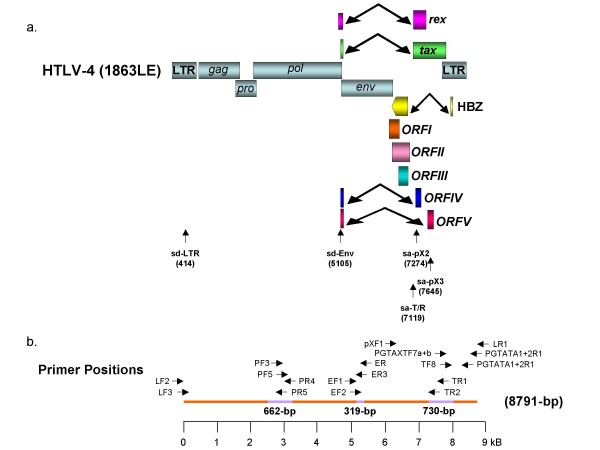
**Organization of the HTLV-4 genome (a) and schematic representation of the PCR-based genome walking strategy (b)**. (a) shown are non-coding long terminal repeats (LTR), coding regions for all major proteins (*gag*, group specific antigen; *pro*, protease; *pol*, polymerase; *env*, envelope; *rex*, regulator of expression; *tax*, transactivator), HTLV basic leucine zipper (HBZ), and 3' genomic open reading frames (ORF) of unknown function. Putative splice donor (sd) and splice acceptor (sa) sites are indicated. (b) Small proviral sequences (purple bars) were first amplified from each major gene region and the long terminal repeat using generic primers as described in methods. The complete proviral sequence was then obtained by using PCR primers located within each major gene region by genome walking as indicated with arrows and orange bars.

**Figure 2:**
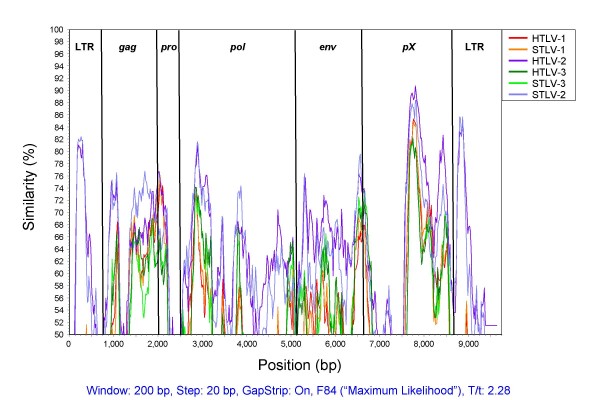
**Similarity plot analysis of the full-length HTLV-4(1863LE) and PTLV genomes using a 200-bp window size in 20 step increments on gap-stripped sequences**. The F84 (maximum likelihood) model was used with a transition-to-transversion ratio of 2.28.

The complete genome of HTLV-4(1863LE) was obtained using a PCR strategy as depicted in Fig. 1 and was determined to be 8791-bp in length. Comparison of the HTLV-4(1863LE) sequence with prototypical PTLV genomes demonstrates that this newly identified human virus is nearly equidistant from HTLV-1 (62% identiity), PTLV-2 (70.7% identity), and PTLV-3 (63.4% identity) groups across the genome (Table 1). The most genetic divergence between HTLV-4 and the other PTLV groups was seen in the LTR (43–65%) and protease (*pro*) gene (59–70%), while the greatest nucleotide identity and amino acid similarity was observed within the highly conserved regulatory genes, *tax *and *rex *(73–81% and 58–91%, respectively). This relationship was highlighted further by comparing HTLV-4(1863LE) with prototypical full-length STLV and HTLV genomes in a similarity plot analysis, where the highest similarity was seen in the highly conserved *tax *gene, which is located at the 5' end of the pX region of the genome (Fig. 2). As seen within other PTLV groups [31], no clear evidence of genetic recombination of HTLV-4(1863LE) with prototypical HTLV and STLV proviral sequences was observed using bootscanning analysis in the SimPlot program (data not shown).

**Table 1:** Percent Nucleotide Identity and Amino Acid Similarity of HTLV4(1863LE) with other PTLV Prototypes^1^.

	HTLV-1 (ATK)	HTLV-2 (MoT)	HTLV-2 (Efe)	STLV-2 (PanP)	STLV-2 (PP1664)	HTLV-3 (2026ND)	STLV-3 (TGE2117)
**Genome**	62.0	70.7	70.8	70.4	70.8	63.2	63.5

**LTR**	45.2	63.2	62.5	62.5	64.7	42.9	45.7

***gag***	70.4 (82.0)	73.2 (85.9)	74.6 (85.9)	74.7 (85.0)	74.8 (86.3)	68.3 (83.6)	68.6 (82.7)
p19M^2^	(68.5)	(77.5)	(79.1)	(79.1)	(79.1)	(78.2)	(71.9)
p24C^2^	(90.2)	(91.6)	(91.6)	(91.5)	(92.6)	(89.3)	(90.2)
p15NC^2^	(81.5)	(84.0)	(81.5)	(81.5)	(84.0)	(76.5)	(74.1)

***pro***	59.0 (61.9)	66.7 (70.8)	66.5 (59.3)	70.0 (59.6)	67.0 (60.0)	64.1 (65.5.)	64.7 (60.0)

***pol***	63.9 (68.0)	71.4 (78.7)	71.5 (78.6)	71.1 (73.3)	71.2 (78.7)	64.9 (71.4)	65.5 (71.2)

***env***	65.8 (75.9)	73.1 (85.3)	72.8 (86.0)	72.0 (84.9)	72.0 (85.5)	68.5 (79.4)	67.2 (78.9)
SU^3^	(70.4)	(80.1)	(81.4)	(80.4)	(81.4)	(72.9)	(71.7)
TM^3^	(84.7)	(94.4)	(93.8)	(92.7)	(92.7)	(90.5)	(91.0)

***rex***	76.0 (63.9)	79.5 (74.1)	81.1 (75.3)	78.7 (65.2)	80.7 (68.8)	72.7 (59.4)	72.5 (57.7)

***tax***	75.9 (82.6)	80.0 (90.9)	80.1 (89.5)	76.7 (85.5)	77.2 (90.0)	74.2 (82.6)	74.1 (82.6)

^1 ^amino acid similarity in parentheses; strain names given in parentheses

^2 ^M, matrix protein; C, capsid; NC, nucleocapsid

^3 ^SU, surface protein; TM, transmembrane

### Phylogenetic analysis

**Figure 3:**
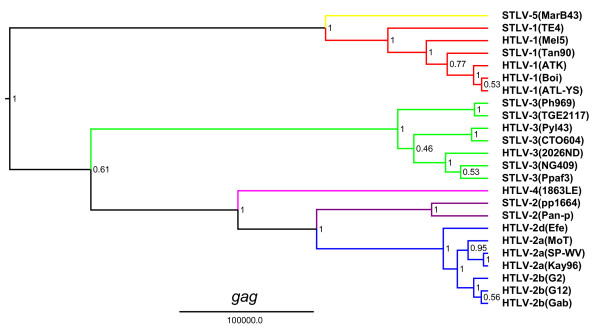
**Phylogenetic relationship of HTLV-4(1863LE) to other PTLVs in *gag *using Bayesian inference**. First and second codon positions of *gag *were used to generate PTLV phylogenies by sampling 10,000 trees with a Markov Chain Monte Carlo method under a relaxed clock model, and the maximum clade credibility tree, i.e. the tree with the maximum product of the posterior clade probabilities, was chosen. Branch lengths are proportional to median divergence times in years estimated from the post-burn in trees with the scale at the bottom indicating 100,000 years. Posterior probabilities for each node are indicated. Branches leading to PTLV-1, HTLV-2 and PTLV-3 sequences are drawn in red, blue and green respectively. The branch leading to HTLV-4(1863LE), STLV-2, and to the divergent MarB43 strain are drawn in magenta, purple, and yellow respectively.

**Figure 4:**
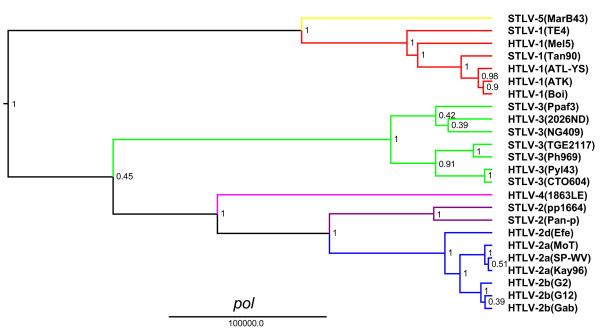
**Phylogenetic relationship of HTLV-4(1863LE) to other PTLVs in *pol *using Bayesian inference**. First and second codon positions of *pol *sequences were used to generate PTLV phylogenies by sampling 10,000 trees with a Markov Chain Monte Carlo method under a relaxed clock model, and the maximum clade credibility tree, i.e. the tree with the maximum product of the posterior clade probabilities, was chosen. Branch lengths are proportional to median divergence times in years estimated from the post-burn in trees with the scale at the bottom indicating 100,000 years. Posterior probabilities for each node are indicated. Branches leading to PTLV-1, HTLV-2 and PTLV-3 sequences are drawn in red, blue and green respectively. The branch leading to HTLV-4(1863LE), STLV-2, and to the divergent MarB43 strain are drawn in magenta, purple, and yellow respectively.

**Figure 5:**
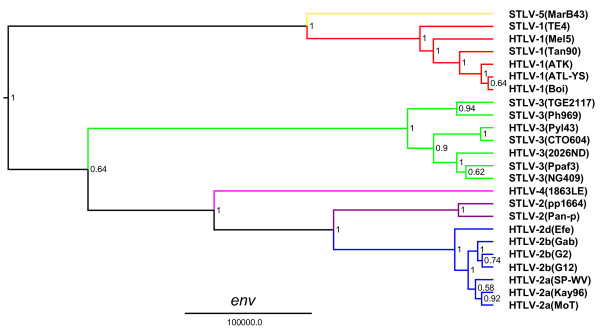
**Phylogenetic relationship of HTLV-4(1863LE) to other PTLVs in *env *using Bayesian inference**. First and second codon positions of *env *sequences were used to generate PTLV phylogenies by sampling 10,000 trees with a Markov Chain Monte Carlo method under a relaxed clock model, and the maximum clade credibility tree, i.e. the tree with the maximum product of the posterior clade probabilities, was chosen. Branch lengths are proportional to median divergence times in years estimated from the post-burn in trees with the scale at the bottom indicating 100,000 years. Posterior probabilities for each node are indicated. Branches leading to PTLV-1, HTLV-2 and PTLV-3 sequences are drawn in red, blue and green respectively. The branch leading to HTLV-4(1863LE), STLV-2, and to the divergent MarB43 strain are drawn in magenta, purple, and yellow respectively.

**Figure 6:**
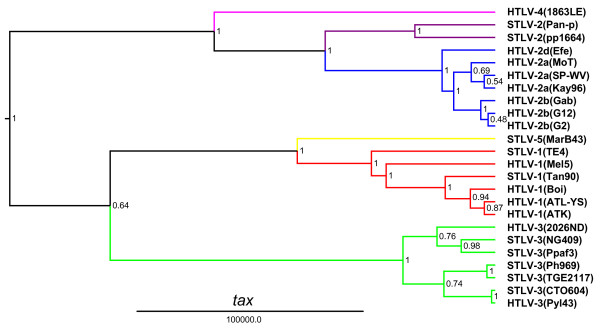
**Phylogenetic relationship of HTLV-4(1863LE) to other PTLVs *tax *using Bayesian inference**. First and second codon positions of *tax *sequences were used to generate PTLV phylogenies by sampling 10,000 trees with a Markov Chain Monte Carlo method under a relaxed clock model, and the maximum clade credibility tree, i.e. the tree with the maximum product of the posterior clade probabilities, was chosen. Branch lengths are proportional to median divergence times in years estimated from the post-burn in trees with the scale at the bottom indicating 100,000 years. Posterior probabilities for each node are indicated. Branches leading to PTLV-1, HTLV-2 and PTLV-3 sequences are drawn in red, blue and green respectively. The branch leading to HTLV-4(1863LE), STLV-2, and to the divergent MarB43 strain are drawn in magenta, purple, and yellow respectively.

The unique genetic relationship of HTLV-4(1863LE) to other PTLVs was confirmed by Bayesian phylogenetic analysis that inferred trees using alignments of each major viral gene in the PTLV genome after excluding 3^rd ^codon positions (cdp) which were significantly saturated as determined by pair-wise transition and transversion versus genetic divergence plots using the DAMBE program (Additional file 1, Fig. S1). At the 3^rd ^cdp transitions and transversions plateaued indicating sequence saturation (Additional file 1, Fig. S1). In contrast, transitions and transversions increased linearly for the 1^st ^and 2^nd ^cdp without reaching a plateau indicating they still retained enough phylogenetic signal (Additional file 1, Fig. S1). Maximum clade credibility trees inferred by using a Markov Chain Monte Carlo (MCMC) sampler showed three major, well supported, monophyletic PTLV groups (posterior probability p = 1.0) with HTLV-1, HTLV-2, and HTLV-3, each clustering in separate clades (Figs. 3, 4, 5 and 6). For each gene region analyzed, HTLV-4 appears as an independent and highly divergent monophyletic lineage sharing a common ancestor with the PTLV-2 clade (p = 1.0). The phylogenetic relationships among PTLV lineages inferred from different gene regions were also similar (Figs. 3, 4, 5 and 6). The only exception was the monophyletic PTLV-3 lineage which was either a sister lineage to PTLV-4/PTLV-2 or PTLV-5/PTLV-1 [10] in the *gag *(Fig. 3) and *env *(Fig. 5) or *pol *(Fig. 4) and *tax *(Fig. 6) tree topologies, respectively, but in each case with weak posterior probabilities (p < 0.75) (Figs 3, 4, 5 and 6). Similarly, the position of the PTLV-3 phylogroup was unresolved using both the maximum likelihood (ML) and Neighbor Joining (NJ) methods (Additional file 1, Fig. S2). The long branch length leading to the HTLV-4 strain suggests an ancient separation of this lineage from PTLV-2. Similarly, STLV-1(MarB43) and STLV-2 each formed distinct lineages from PTLV-1 and HTLV-2, respectively, with long branch lengths (Figs. 3, 4, 5 and 6). These findings support further the recent re-classification of STLV-1(MarB43) as a new PTLV lineage called STLV-5 and the need to re-classify STLV-2 as a distinct PTLV group [10]. The unequivocal monophyletic relationship of HTLV-4 to other PTLVs was supported further by phylogenetic inference of similar tree topologies with robust statistical support obtained with NJ and ML analysis, using both separate alignments for each genes and the full-length genome without LTRs (Additional file 1, Fig. S2).

### Dating the origin of HTLV-4(1863LE) and other PTLVs

The long branch leading to the HTLV-4 strain suggests an ancient, independent evolution of this human retrovirus. Hence, additional molecular analyses were performed to estimate the divergence times of the HTLV and PTLV lineages. Although we and others have reported finding a clock-like behavior of PTLV sequences using partial LTR or *env *sequences [3,18-20], we were unable to confirm these results. Instead, the clock hypothesis was strongly rejected (p < 0.00001) for the 1^st ^+ 2^nd ^codon position alignment of full-length PTLV genomes without LTRs, as well as for separate alignments of full-length *gag*, *pol*, *env *and *tax *genes (p < 0.00001 in each case) suggesting significant evolutionary rate heterogeneity among the different viral lineages. Indeed, sequence analysis showed unequal base composition for some lineages and substitution saturation at the 3^rd ^codon position (cdp) for all PTLVs (Additional file 1, Fig. S1). Substitution saturation was not observed in the 1^st ^and 2^nd ^cdps (Additional file 1, Fig. S1) and these sites were thus suitable for estimating posterior evolutionary rates and divergence dates of PTLV by using Bayesian analysis with a MCMC algorithm.

The relaxed molecular clock was calibrated with two independent molecular calibration points; 12,000 – 30,000 ya as confidence intervals for the origin of HTLV-2 as it migrated out of Africa and Asia and into the Americas via the Bering land bridge and 40,000 – 60,000 ya as confidence intervals for the origin of HTLV-1 in Melanesia as it became populated with people from Asia [23,32,33]. The use of two calibration points has previously been shown to provide more reliable estimates of PTLV substitution rates than a single calibration date [3,32]. Using these methods we found that the PTLV posterior mean evolutionary rates differed for each of the four major coding regions and ranged from 2.89 × 10^-7 ^to 7.92 × 10^-7 ^substitutions/site/year (Table 2). The highest mean evolutionary rate was seen in *pol *while the lowest rate was observed in *gag *(Table 2). These rates are consistent with those calculated previously using the same calibration points with and without enforcing a molecular clock [3,4,18-20,23,31,32], including those of Lemey *et al. *who also found disparate PTLV evolutionary rates across the PTLV genome [33].

**Table 2:** PTLV evolutionary rates^1 ^at 1^st ^+ 2^nd ^codon positions of different gene regions assuming a Bayesian relaxed molecular clock.

Gene region	α-parameter (Γ-distribution)^2^	Mean rate	Median rate	95% HPD
*gag*	0.23 (0.168 – 0.303)	3.02 × 10^-7^	2.89 × 10^-7^	1.65 – 4.78 × 10^-7^
*pol*	0.417 (0.356 – 0. 475)	7.92 × 10^-7^	7.57 × 10^-7^	3.93 – 12.7 × 10^-7^
*env*	0.29 (0.228 – 0.359)	4.08 × 10^-7^	3.9 × 10^-7^	2.25 – 6.44 × 10^-7^
*tax*	0.311 (0.215 – 0.421)	4.32 × 10^-7^	4.17 × 10^-7^	2.34 – 6.47 × 10^-7^

^1 ^Evolutionary rate is in nucleotide substitutions/site/year.

^2 ^95% HPD (high posterior density) intervals are given in parentheses.

Median estimates and 95% high posterior density (95% HPD) intervals for the time of the most recent common ancestor (tMRCA) of the major PTLV clades according to different gene regions are given in Table 3. The tMRCA of the PTLV tree ranged between 214,650 (*tax *gene) and 385,100 ya (*env *gene) confirming an ancient evolution of the primate deltaretroviruses [3]. These dates are lower than those reported previously for the PTLV cenancestor which were inferred using methods less accurate than the Bayesian analyses employed here [3,4]. Remarkably, the inferred PTLV divergence dates were very similar for each gene region with those estimated for the highly conserved *tax *gene being slightly lower (Table 3). Nevertheless, the 95% HPD intervals overlapped for all four genes (Table 3) supporting the strength of the inferred PTLV divergence dates. Estimates for the PTLV-4 progenitor split from PTLV-2 ranged between 124,250 ya (c.i., 49,800 – 218,250 ya) in the *tax *gene to 221,650 ya (c.i., 89,650 – 378,000 ya) in the *env *gene and were comparatively earlier than the median tMRCA of PTLV-1 (54,250–75,100 ya), PTLV-2 (75,200–128,600 ya), and PTLV-3 (40,850–71,700 ya) clades (Table 3). These results suggest that the HTLV-4/PTLV-2 ancestor may represent the oldest PTLV identified to date.

**Table 3:** PTLV evolutionary time-scale calculated with a Bayesian relaxed molecular clock using 1^st ^+ 2^nd ^codon positions of different gene regions^1^.

Clade	*gag*	*pol*	*env*	*tax*
PTLV root	358,500 (169,200 – 600,200)	308,500 (136,400 – 559,900)	385,100 (172,300 – 638,900)	214,650 (104,050 – 353,100)
				
STLV-5 (MarB43)/PTLV-1	121,850 (68,650 – 201,300)	121,450 (60,450 – 220,600)	147,850 (72,450 – 244,800)	87,500 (50,400 – 143,250)
PTLV-1	75,100 (50,200 – 115,200)	54,250 (40,410 – 79,340)	58,250 (41,600 – 84,000)	54,800 (40,900 – 76,100)
HTLV-1(Mel)/PTLV1a, b ^2^	46,350 (40,000 – 57,900)	47,450 (40,000 – 58,400)	47,550 (40,000 – 58,400)	48,200 (40,000 – 58,500)
				
**HTLV-4(1863)/PTLV-2**	**187,500** **(85,050 – 321,800)**	**175,100** **(63,850 – 334,750)**	**221,650** **(89,650 – 378,000)**	**124,250** **(49,800 – 218,250)**
PTLV-2	128,600 (57,000 – 226,550)	103,700 (41,300 – 205,100)	126,850 (51,850 – 223,350)	75,200 (29,850 – 135,200)
STLV-2	42,350 (11,650 – 87,100)	37,200 (9,800 – 82,800)	27,700 (8,150 – 58,100)	35,550 (12,100 – 70,050)
HTLV-2	33,600 (15,750 – 58,200)	30,100 (13,900 – 54,900)	30,600 (13,750 – 54,100)	23,500 (12,800 – 41,050)
HTLV-2a, b ^3^	23,000 (14,350 – 30,000)	20,400 (12,000 – 28,700)	20,000 (12,000 – 28,350)	18,350 (12,000 – 27,950)
				
PTLV-3	71,700 (28,800 – 120,700)	64,550 (25,010 – 129,800)	60,050 (32,950 – 122,200)	40,850 (16,400 – 81,150)

^1 ^The most recent common ancestor (tMRCA) is the median Bayesian estimate in years ago (ya); 95% HPD intervals are given in parentheses.

^2 ^The tMRCA for this node was constrained by using a uniform distribution prior of 40,000–60,000 ya.

^3 ^The tMRCA for this node was constrained by using a uniform distribution prior of 12,000–30,000 ya.

### Genomic organization and characterization of the HTLV-4(1863LE) structural and enzymatic proteins, and the LTR

**Figure 7:**
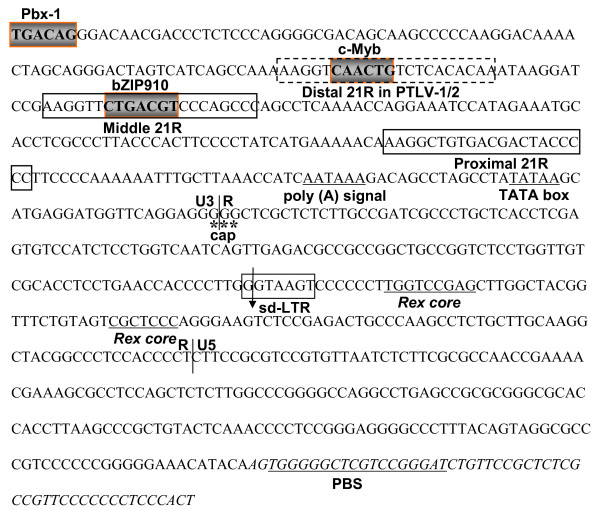
**Nucleotide sequence of the HTLV-4(1863LE) LTR and pre-*gag *region**. The U3-R-U5 locations (vertical lines), the pre-B cell leukemia (Pbx-1, TGACAG) and c-Myb (YAACKG) transcription factor binding sites, approximate cap site (cap), polyadenylation (poly(A)) signal, TATA box, predicted splice donor site (sd-LTR), and two 21-bp repeat elements (middle and proximal based on positions in HTLV-1 and -2), as well as the location of the distal 21-bp repeat in HTLV-1 and -2 (dashed lines), are indicated. In the R and U5 regions, the predicted Rex core elements and nuclear riboprotein A1 binding sites are underlined. The pre-*gag *region and primer binding site (PBS, underlined) are in italics.

The genomic structure of HTLV-4(1863LE) was similar to that of other PTLVs and included the structural, enzymatic, and regulatory proteins all flanked by long terminal repeats (LTRs) (Fig. 1). Like HTLV-3 (697-bp), the HTLV-4(1863LE) LTR (696-bp) was smaller than that of HTLV-1 (756-bp) and HTLV-2 (764-bp), by having two rather than the typical three 21-bp transcription regulatory repeat sequences in the U3 region of HTLV-1 and HTLV-2 (Fig. 7) [18-20,23,31,34,35]. The distal 21-bp repeat element found in HTLV-1 and HTLV-2 is absent from the HTLV-4(1863LE) genome (Fig. 7). Others have shown that deletion of the middle, rather than the distal 21-bp element, is more critical for the loss of basal HTLV-1 transcription levels [36]. In addition, the lack of the distal 21-bp repeat does not seem to affect viral expression of PTLV-3 [35,37]. Nonetheless, additional studies are needed to determine what effect the absence of a 21-bp element has on HTLV-4(1863LE) gene expression and replication.

**Figure 8:**
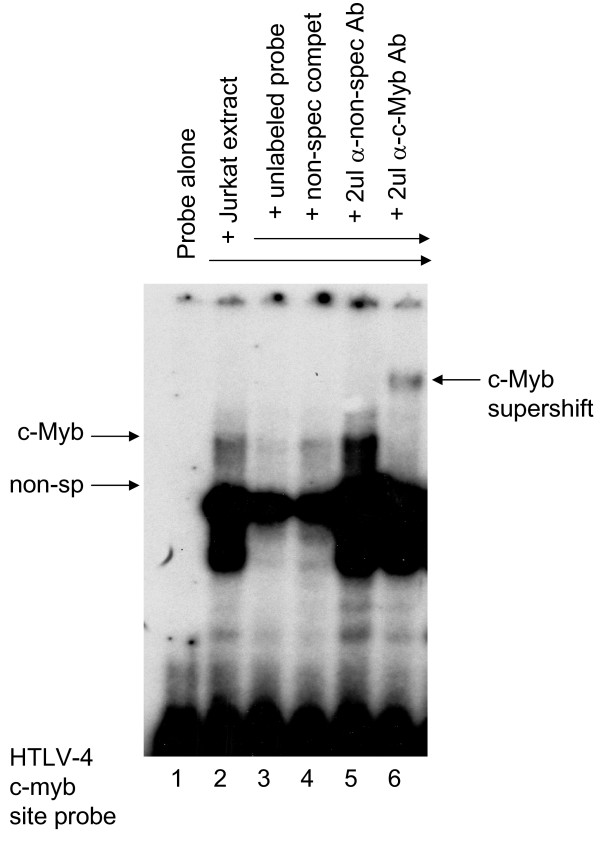
**EMSA using a ^32^P-labeled probe representing the c-Myb binding site within the HTLV-4 LTR (lane 1) incubated with Jurkat nuclear extract (lanes 2–6)**. A 100-fold excess of unlabeled probe sequence (specific competitor, lane 3) or an unlabeled oligonucleotide containing mutations within the c-Myb binding site (non-specific competitor, lane 4) were added as indicated. Non-specific (lane 5) and Myb-specific (lane 6) antibodies were added and the supershifted band is indicated on the right panel, which is a longer exposure of the left panel.

**Figure 9:**
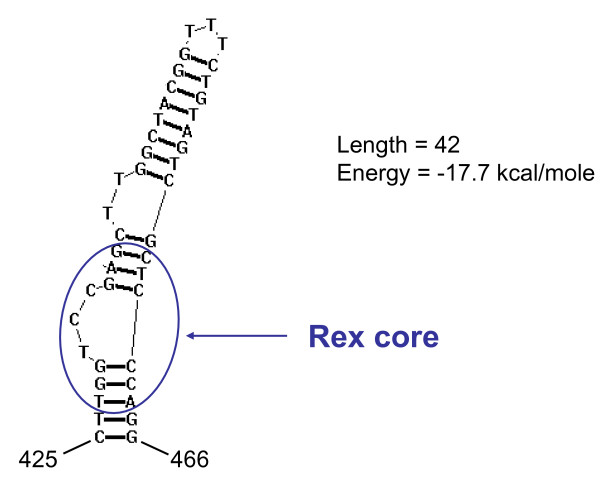
**Plot of predicted RNA stem loop secondary structure of HTLV-4(1863LE) LTR region**. Position of the Rex responsive element (RexRE) core is indicated.

Other regulatory motifs such as the polyadenylation signal, TATA box, and cap site were all conserved in the HTLV-4(1863LE) LTR (Fig. 7). Highly conserved pre-B cell leukemia (Pbx-1, TGACAG) and c-Myb (YAACKG) transcription factor binding sites were also identified at positions 1–6 and 86–91 of the LTR, respectively, upstream of the first 21-bp repeat element (Fig. 7). The Pbx-1 and c-Myb sites are also conserved in the LTRs of STLV-2 and two nearly identical PTLV-3 strains (STLV-3(CTO604) and HTLV-3(Pyl43)) [15,16,19,34], respectively, but are absent in other PTLV LTRs. Binding to the predicted c-Myb target sequence within the HTLV-4 LTR oligonucleotide was observed and was specific based upon banding patterns observed in the presence of specific and non-specific oligonucleotide competitors in an electrophoretic mobility shift assay (EMSA). The shifted band was identified as c-Myb since an anti-c-Myb antibody supershifted the complex while an unrelated antibody did not (Fig 8). While this analysis confirms the specificity of the putative c-Myb binding site in the HTLV-4 LTR oligonucleotide and likely reflects binding of c-Myb to the HTLV-4 LTR, this remains to be tested *in vivo*. Secondary structure analysis of the LTR RNA sequence predicted a stable stem loop structure from nucleotides 425 – 466 (Fig 9) similar to that shown to be essential for Rex-responsive viral gene expression in both HTLV-1 and HTLV-2.

Translation of predicted protein open reading frames (ORFs) across the viral genome identified all major Gag, Pro (protease), Pol, and Env proteins, as well as the regulatory proteins, Tax and Rex (Fig. 1). Translation of the overlapping *gag *and *pro *and *pro *and *pol *ORFs occurs by one or more successive -1 ribosomal frameshifts that align the different ORFs. The conserved slippage nucleotide sequence 6(A)-8nt-6(G)-11nt-6(C) is present in the Gag-Pro overlap starting at nucleotide 1997. Similarly, the Pro-Pol overlap slippage sequence (TTTAAAC) was identical to that seen in HTLV-1 and HTLV-2 but which is different from that found in HTLV-3 by a single nucleotide substitution at the beginning of this motif (**G**TTAAAC) [31]. Importantly, the asparagine codon (AAC) crucial for the slippage mechanism is conserved in all HTLVs.

The structural and group-specific precursor Gag protein consisted of 424 amino acids (aa), and is predicted to be cleaved into the three core proteins p19 (matrix), p24 (capsid), and p15 (nucleocapsid) similar to HTLV-1, HTLV-2, and HTLV-3. Across PTLVs, Gag is one of the most conserved proteins, with the HTLV-4 Gag having 82% to 86% similarity to HTLV-1, PTLV-2, and PTLV-3 (Table 1). The Gag capsid protein (214 aa) showed about 90% to 93% similarity to other PTLV capsids, while the matrix (129 aa) and nucleocapsid (81 aa) proteins were somewhat less conserved, showing less than 85% similarity to HTLV-1, PTLV-2, and PTLV-3 (Table 1). The conservation of the capsid protein supports the observed cross-reactivity to Gag seen with plasma from the HTLV-4-infected person in Western blot (WB) assays employing HTLV-1 antigens [6,38].

The predicted size of the HTLV-4 (1863LE) Env polyprotein is 485 aa, which is slightly shorter than the Env of PTLV-2 (486 aa), PTLV-1 (488 aa), and PTLV-3 (491–492 aa). The Env surface (SU) protein (307 aa) showed the most genetic divergence from other PTLVs with only 70% – 81% similarity, while the transmembrane (TM) protein (178 aa) was highly conserved across all PTLVs, sharing 85% – 94% similarity, supporting the use of recombinant HTLV-1 TM protein (GD21) on WB strips to identify divergent PTLVs, including HTLV-4. The HTLV-4(1863LE) SU showed about 86% similarity to the HTLV-2 type specific SU peptide (K55) despite the observed weak reactivity of anti-HTLV-4(1863LE) antibodies to [6,38] K55 spiked onto WB strips. This amino acid similarity is somewhat greater than the 67.4% and 72.1% similarity of the HTLV-1 and HTLV-3 SUs to K55, respectively, allowing serologic discrimination of HTLV-2 from HTLV-1 in this region. In contrast, the HTLV-4(1863LE), HTLV-2, and HTLV-3 SUs share from 68.8% to 70.8% similarity to the HTLV-1 type specific SU peptide (MTA-1). Although these results are limited to testing the sera of a single HTLV-4-infected individual, they suggest that higher antibody reactivity to the HTLV-2-type specific peptide may be observed in HTLV-4-infected persons [38].

The glucose transporter GLUT1 has been shown to be the HTLV-1 and -2 envelope receptor and a retrovirus binding domain (RBD) for GLUT1 has been identified in the SU of these viruses [39,40]. Analysis of the HTLV-4 Env protein revealed a putative RBD located at positions 85 – 138 of the SU that shared about 80%, 78%, and 87% amino acid similarity with the RBDs of HTLV-1(ATK), HTLV-2(MoT), and that identified by analysis of the HTLV-3(2026ND) Env, respectively. In addition, both aspartic acid and the tyrosine residues located as positions 106 and 114 of HTLV-1(ATK) are highly conserved in the putative HTLV-4 RBD and all other PTLV RBDs (data not shown), supporting a critical role for these residues as the receptor binding core as previously suggested [41].

### Characterization of Regulatory and Accessory Proteins of HTLV-4(1863LE)

**Figure 10:**
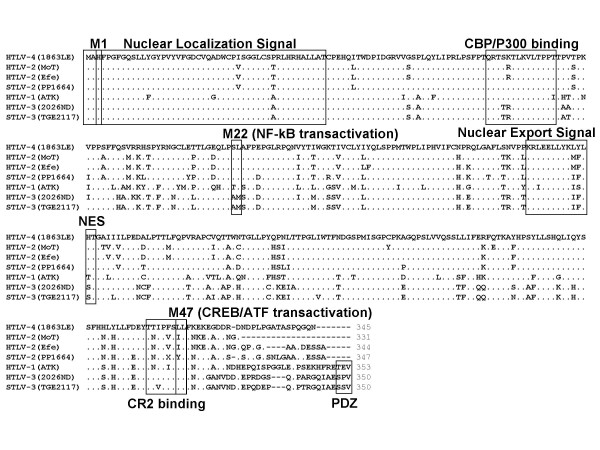
**Comparison of predicted Tax amino acid sequences of selected prototypical primate T-cell lymphotropic viruses**. Shown in boxes are known functional motifs: NLS, nuclear localization signal; (CBP)/P300, cAMP response element (CREB) binding protein; NES, nuclear export signal; CR2, C-terminal transcriptional activating domain; PDZ, PDZ binding motif; M1, M22, and M47 are motifs important for Tax transactivation and NF-kβ activation (38).

The HTLV-1, HTLV-2, and HTLV-3 Tax proteins (Tax1, Tax2, and Tax3, respectively) transactivate initiation of viral gene expression from the promoter located in the 5' LTR and are thus essential for viral replication [27,30,42]. Tax1 and Tax2 have also been shown to be important for T-cell immortalization [27,30]. To characterize the HTLV-4 Tax (Tax4) we compared the sequence of Tax4 with those of prototypic HTLV-1, PTLV-2, and PTLV-3s to determine if motifs associated with specific Tax functions were preserved between each group. Alignment of the predicted Tax4 sequence shows excellent conservation of the critical functional regions, including the nuclear localization signal (NLS), cAMP response element (CREB) binding protein (CBP)/P300 binding motifs, and nuclear export signal (NES) (Fig. 10). Three sets of amino acids (M1, M22, M47) shown to be important for Tax1 transactivation and activation of the nuclear factor (NF)-kβ pathway are also highly conserved in Tax4 (Fig. 10) [43]. The C-terminal transcriptional activating domain (CR2), essential for CBP/p300 binding, was also conserved within Tax4, except for two mutations, N to T and I/V to F, at positions two and five of the motif, respectively (Fig. 10). However, the CR2 binding domain of the STLV-3 Tax, which contains these identical mutations, has been shown recently to retain its ability to bind CBP and to a lesser extent p300 with no deleterious effect on transactivation of the viral promoter [42].

Although important functional motifs are highly conserved in PTLVs, phenotypic differences between HTLV-1 and HTLV-2 Tax proteins have lead to speculation that these differences account for the different pathologies associated with both HTLVs [27]. Recently, the C-terminus of Tax1, but not Tax2, has been shown to contain a conserved PDZ binding domain present in cellular proteins involved in signal transduction and induction of IL-2-independent growth required for T-cell transformation [29,44,45] and may contribute to the phenotypic differences between these two viral groups. The consensus PDZ domain has been defined as S/TXV-COOH, where the first amino acid is serine or threonine, X is any amino acid, followed by valine and the carboxyl terminus. Tax4 does not contain a PDZ domain (Fig. 10), suggesting that like HTLV-2, HTLV-4 may possibly be less pathogenic than HTLV-1.

**Figure 11:**
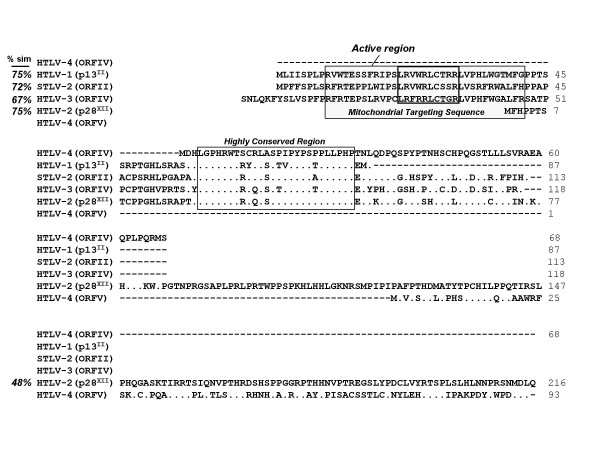
**Comparison of predicted accessory protein sequences of selected primate T-cell lymphotropic viruses**. Upper alignment, HTLV-4(1863LE) open reading frame (ORF) IV compared to HTLV-1 (p13^II^), STLV-2 ORFII, HTLV-3(2026ND) ORFIV, and HTLV-2 p28^XII^. Location of conserved mitochondrial targeting sequence in the HTLV-1 p13^II ^protein and highly conserved amino acid region are boxed. Lower alignment, HTLV-4(1863LE) ORFV compared to HTLV-2 ORFII (p28^XII^). % Sim, percent amino acid similarity of  HTLV-4 ORFs to other PTLV ORFs.

Besides Tax and Rex, two additional ORFs encoding four proteins, p27^I^, p12^I^, p30^II^, and p13^II ^(where I and II denote ORFI and ORFII, respectively), have been identified in the pX region of HTLV-1 and are important in viral infectivity and replication, T-cell activation, and cellular gene expression [26]. Analysis of the pX region of HTLV-4(1863LE) revealed a total of five additional putative ORFs (named I-V, respectively) encoding predicted proteins of 101, 161, 99, 133, and 115 aa in length (Fig 1a). Since none of the potential ORFs begin with methionine start codons, we determined potential splice junctions in the HTLV-4 genome to ascertain the potential for novel ORFs via complex splicing mechanisms. Prediction of splice junction positions in HTLV-4 identified only two donor sites with high confidence, one at nucleotide 414 in the LTR (sd-LTR) and one at nucleotide 5105 in Env (sd-Env) (Fig. 1a). Three additional putative splice acceptor sites were identified at nucleotides 7274 (sa-pX2) and 7645 (sa-pX3), and in Tax/Rex at nucleotide 7245 (sa-T/R). The sa-T/R is used with the sd-Env to generate the Tax and Rex proteins via complex splicing mechanisms (Fig. 1). Rex mRNA is predicted to be spliced using sd/sa sites in a different reading frame than Tax and with a different methionine start codon (nucleotide positions 5043 – 5105 and 7120 – 7566) to generate a 170 aa protein. Tax mRNA is spliced from nucleotide positions 5102 – 5105 and 7120 – 8150 to generate a protein predicted to be 345 aa in length. Two potential accessory proteins 68 and 93 aa in length are then predicted using the sd-Env and either the sa-pX2 or sa-pX3 in ORFIV or ORFV, respectively (Figs. 1 and 11). The HTLV-4 ORFIV protein shared 75% similarity with the HTLV-1p13^II ^and HTLV-2 p28^II ^accessory proteins but was missing the mitochondrial targeting sequence and the active region typically located at the amino-terminus of the protein (Fig. 11). Interestingly, 19 of 26 (73%) amino acids in the HTLV-4 ORFIV (positions 4–29) were identical to similar ORFs from all other major PTLVs, suggesting a conserved functionality of this motif (Fig. 11). The predicted HTLV-4 ORFV protein shared only weak similarity (41%) to the carboxyl-terminus of the HTLV-2 p28X^II ^protein (Fig. 11). In contrast to the HTLV-4 ORFIV and ORFV proteins, the predicted HTLV-4 ORFI, ORFII, and ORFIII proteins did not share significant sequence identity with any PTLV accessory proteins, but shared weak sequence similarity with only miscellaneous microbial proteins available in GenBank such as *Pseudomonas *histidine kinase (37% similarity) (data not shown). Analysis of alternatively spliced messenger RNA expression in viable cells or tissue culture, and/or in vitro characterization, will be required to investigate the expression and functionality of these putative accessory proteins.

**Figure 12:**
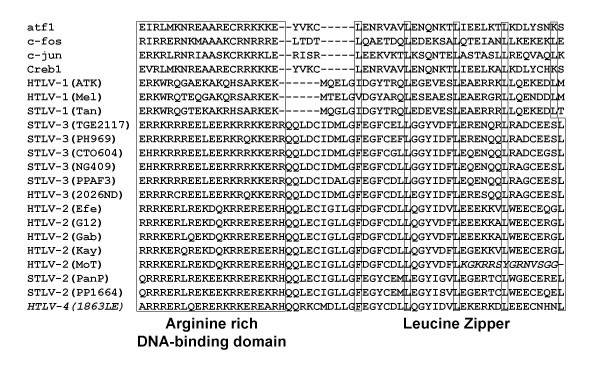
**Comparison of predicted amino acid sequences of primate T-cell lymphotropic viruses and cellular basic leucine zipper (bZIP) transcription factors**. Conserved arginine rich and potential leucine zipper regions of the bZIP proteins are boxed. Alternate amino acid sequence resulting from frameshift mutation in HTLV-2(MoT) leucine zipper region is shown in italics.

A novel protein termed the HTLV-1 basic leucine zipper ZIP (bZIP) factor (HBZ) was recently found to be encoded on the complementary strand of the viral RNA genome between the *env *and *tax/rex *genes which was shown to negatively regulate viral replication and to enhance viral infectivity and persistence [28,46]. The recent finding of HBZ mRNA as the sole viral gene product expressed in ATL patients also suggests a role of HBZ mRNA in the survival of leukemic cells *in vivo *and in HTLV-1-associated oncogenesis [47]. Although originally reported to be exclusive to PTLV-1 [28], we previously reported that HBZ is conserved among PTLV-1, -2, and -3 [31]. More recently, others have demonstrated that an HTLV-3(Pyl43) molecular clone expressed an antisense mRNA [48]. Although these results confirm the predicted HBZ gene region in this virus [34], additional studies are required to evaluate the functionality of the HTLV-3 HBZ protein. We now show by sequence analysis that an HBZ homolog is also present in HTLV-4 emphasizing the potential importance of this protein and mRNA in viral replication, persistence, and leukemogenesis [28,46]. The carboxyl terminus of the HBZ ORF contains a 21 aa arginine rich region that is relatively conserved in PTLV and known cellular bZIP transcription factors, followed by a less conserved leucine zipper region that possesses five or four highly conserved leucine heptads in HTLV-1 and all other PTLVs, respectively (Fig. 12). HTLV-1 has five leucine heptads similar to that found in mammalian bZIP proteins, while all other PTLVs, including PTLV-4, have four leucine heptads followed by leucine octet (Fig. 12). In PTLVs, the first residue in the initial leucine heptad is a nonpolar amino acid other than leucine (Fig. 12). This single amino acid substitution has not affected the functionality of the leucine zipper in HTLV-1 but requires further study of its affect in other PTLV HBZs [25,41]. As reported previously, HTLV-2(MoT) is the only PTLV-2 strain that does not have the full complement of leucine heptads a result of a single nucleotide deletion at position 6823 that causes a frameshift in the predicted HBZ sequence [31].

## Discussion

Here we report the first complete nucleotide sequence and genomic characterization of the recently discovered HTLV-4. We show that the genome of this novel human virus is genetically equidistant from HTLV-1, HTLV-2, and HTLV-3. Robust phylogenetic and molecular clock analysis confirms that HTLV-4 clearly falls outside the diversity of PTLV-1, PTLV-2, and PTLV-3, demonstrating that HTLV-4 is the only known member of a distinct PTLV group we call PTLV-4. Combined, these results strongly support the HTLV-4/PTLV-4 nomenclature proposed for this virus [6]. The phylogenetic stability seen across HTLV-4 and other PTLV genomes also demonstrates the absence of major recombination events occurring in PTLV despite evidence of dual infections in humans and primates [9,49]. Furthermore, these results support the distinct evolutionary history of HTLV-4 and other PTLVs demonstrating that they are not recent genetic recombinants from pre-existing viral genomes. This finding contrasts with other retroviruses like HIV in which frequent recombination contributes substantially to genetic diversity [50].

Bayesian MCMC statistical methods have recently been developed to accurately infer dates of evolutionary events, to investigate the origin of viral epidemics, and to estimate historical population dynamics [32,51]. Molecular dating of the HTLV-4 predecessor using these robust methods suggests that this novel PTLV lineage originated almost 200 millennia ago, which predates the inferred origin of the ancestors of HTLV-1, HTLV-2, and HTLV-3 by about 76,000 – 191,000 ya [31]. Two equally parsimonious hypotheses on the origin of HTLV-4 can thus be proposed by the inferred ancient existence of the PTLV-4 lineage. First, it is possible that HTLV-4(1863LE) is a current descendent of the ancestral PTLV-4 that infected humans as they evolved in Africa and represents a strain circulating within humans living in this geographic region. Interestingly, the inferred date of the HTLV-4 ancestor also coincides with the appearance of *Homo sapiens sapiens*, estimated to have occurred around 200 – 400 K ya, suggesting the emergent human lineage may have been a suitable host for the ancestral PTLV-4. If this is not just an evolutionary historical coincidence of both virus and host, then HTLV-4 may indeed be the oldest human deltaretrovirus as inferred from the molecular dating of all four HTLV groups. Alternatively, HTLV-4(1863LE) could also be the result of a more recent zoonotic infection with a very divergent STLV present in NHPs in the forests of Cameroon. Additional information on the diversity of HTLV-4 and its likely simian counterpart will be needed to determine whether HTLV-4(1863LE) truly originated as *H. sapiens sapiens *evolved, and persists in humans today, or represents a more recent zoonotic transmission from an NHP. As of yet, a simian counterpart of HTLV-4 has not been identified in Cameroon or elsewhere despite the identification of other novel STLVs in this region [9,10,22]. Nonetheless, the inability to find "STLV-4" may be due to sampling and screening biases in the selection of NHP species and the geographic locations examined [9,32].

The inference of an ancient split of HTLV-4(1863LE) from the PTLV-2 lineage, combined with the wide geographic distribution of STLVs and a history of STLVs crossing into humans [2,8-10,18-21], all imply that HTLV-4 infection may be more prevalent. Repeated and historical cross-species infections of humans with various STLV-1 strains led to the emergence and dissemination of several HTLV-1 subtypes in West-Central Africa [2,4-6]. Similar evidence suggests that the newly identified HTLV-3 infections also potentially arose from multiple, independent past or contemporary introductions of different STLV-3 strains into humans [6,8,31]. Given that both HTLV-1 and HTLV-2 followed human population migrations out of Africa and across the globe as humans evolved, HTLV-4 and HTLV-3 may also have spread globally. A more precise determination of the origin and distribution of HTLV-4 infection will require further studies, such as expanded surveillance in both humans and NHPs. However, serosurveys for HTLV-4 may be complicated by the inability to discriminate this infection from HTLV-2 since they both show similar WB profiles and the sensitivity of serological assays for identifying HTLV-4 is currently unknown [6,35]. Thus, additional diagnostic tools are required to determine the level of HTLV-4 penetration into the general population and to search for the potential primate origin of HTLV-4(1863LE). Screening for HTLV-4 will be facilitated by the development and application of serologic and molecular assays based on the sequences reported here. For example, since the HTLV-4 Gag matrix and nucleocapsid and the envelope surface proteins are divergent from PTLV-1, PTLV-2, and PTLV-3 it may be possible to use them in serologic assays to differentiate the four PTLV groups.

Virus classification is a topic of ongoing discussion and suggestions for nomenclature are typically based on lumping or splitting of taxa into distinct groups. Deltaretrovirus species are classified by the International Committee on Taxonomy of Viruses (ICTV) by differences in genome sequence and viral oncogenes, antigenic properties, natural host range, and pathogenicity. For example, HTLV-1 and HTLV-2 are distinguished mostly by phylogenetic diversity and variable disease outcomes of each virus. Recently, a new deltaretrovirus species, STLV-5, was proposed based on limited analyses of small *tax/rex *sequences from a *Macaca arctoides *(strain MarB43) that was originally classified as STLV-1 [4,10]. Herein, we show by using robust phylogenetic analysis of major coding regions and complete viral genomes that expansion of the current PTLV nomenclature from four to six putative major taxonomic species or groups should be considered. Our natural classification of PTLV groups is based on rigorous phylogenetic inference that demonstrates with high confidence the formation of very distinctive monophyletic lineages outside the diversity of all known viral groups, combined with genetic distances demonstrating the putative new lineage is nearly equidistant from all previously characterized groups, and the placement of the new PTLV groups near the root of the PTLV phylogeny. The first four PTLV phylogroups consist of HTLV-1/STLV-1, HTLV-2, HTLV-3/STLV-3, and HTLV-4. We confirm the existence of the putative STLV-5(MarB43) lineage, while the sixth group consists of the STLV-2(PanP) and STLV-2(PP1664) viruses. However, for simplicity we suggest maintaining the STLV-2 nomenclature historically used for this particular viral group. Each proposed new viral group clearly falls outside the diversity of their nearest PTLV relatives (PTLV-1 and HTLV-2, respectively), is monophyletic with strong bootstrap support and posterior probabilities, and are all roughly genetically equidistant from other PTLVs, and hence should all be classified as distinct viral species. As with all viral nomenclature, PTLV classification as proposed here will require approval of ICTV.

In addition to understanding viral evolutionary history, analysis of full-length genomes can also provide basic information on the replication and pathogenic potential of new viruses. Thus, we examined in detail the genetic structure and sequence of HTLV-4 to determine if important functional motifs involved in viral expression and HTLV-induced leukemogenesis are preserved [26-30,44]. All enzymatic, regulatory, and structural proteins are well conserved in HTLV-4(1863LE), including conserved functional motifs in Tax that are important for viral gene expression and T-cell proliferation, suggesting HTLV-4 is replication competent. We also observed several important molecular features of the HTLV-4 genome involved in viral expression and pathogenicity that are either similar or distinct from other HTLVs. For example, the absence of a PDZ domain in the Tax protein of HTLV-4(1863LE), known to be important in cellular signal transduction and T-cell transformation [29-31], is similar to what is seen in HTLV-2 but not in HTLV-1 and HTLV-3 [27]. The absence of PDZ suggests that the HTLV-4 Tax may be more phenotypically similar to the HTLV-2 than the HTLV-1 Tax. Furthermore, the high amino acid identity of the Tax4 and Tax2 proteins also suggests that Tax4 may function similarly to Tax2 [27]. However, whether the absence of a PDZ domain in HTLV-4 is associated with an absence of specific cellular and/or clinical outcomes like HTLV-2 will require further investigation.

We also identified unique putative c-Myb and Pbx-1 transcription factor binding sites in the U3 region of the LTR of HTLV-4(1863LE). c-Myb is a proto-oncogene that is expressed in T cells induced by mitogen or antigenic stimulation and is involved in cell cycle progression and proliferation of T lymphocytes, such that continuous deregulation of cell cycling may play a role in leukemogenesis [52]. c-Myb has been shown to bind to the HTLV-1 and feline leukemia virus LTRs to increase viral transcription [53,54]. Like c-Myb, dysregulation of the homeoprotein Pbx-1 can also increase leukemogenesis by disturbing hematopoiesis [55]. We demonstrate here that the potential c-Myb binding site in the HTLV-4 LTR specifically binds c-Myb, suggesting that it may also promote LTR-mediated viral expression and which may help overcome the loss of the distal 21-bp repeat element observed in the HTLV-4 LTR. For example, Pbx-1 has been demonstrated to up-regulate transcription of another retrovirus, murine leukemia virus (MuLV), by binding to conserved Pbx-1 transcription factor sites present in MuLV LTRs [56]. The presence of putative c-Myb and Pbx-1 binding sites in the HTLV-4 LTR may provide novel mechanisms of transcriptional control at both the viral and cellular levels not previously known for HTLV. Nevertheless, involvement of the putative novel binding sites in viral transcription and leukemogenesis will require additional studies.

Although originally reported to be exclusive to HTLV-1 [28], we now provide additional evidence for a putative HBZ region among all PTLVs, including HTLV-4(1863LE). Despite the absence of canonical bZIP domains, preliminary experiments show that proteins are transcribed from the HTLV-3, and -4 antisense mRNAs and all were potent inhibitors of Tax induction of HTLV LTR activity with similar cellular localizations like that of the HTLV-1 HBZ (unpublished data). These results not only confirm the predicted HBZ sequences and proteins in these viruses but also demonstrate the potential importance of HBZ in PTLV replication. The finding of a potential bZIP region on the antisense strand of all PTLV genomes also indicates that the nomenclature for this protein should be renamed from HBZ to AEP for antisense encoding protein as suggested [48]. The potential role of AEP in HTLV-induced oncogenesis may be less clear since HTLV-1 and HTLV-2 infection result in different clinical outcomes, while pathologies for HTLV-3 and HTLV-4 have not yet been reported. Additional studies are required to confirm the potential effect of the predicted AEP transcripts and proteins on HTLV-4 and PTLV expression and any role they may have on leukemogenesis.

## Conclusions

The novel HTLV-4 genome independently evolved from an ancient deltaretrovirus lineage and contains many of the functional motifs important for viral expression and possibly oncogenesis, including two novel transcription factor binding sites in the LTR. More studies are needed to further characterize the unique molecular features of HTLV-4 identified here, and to determine whether HTLV-4 is endemic and pathogenic in humans to better understand the public health importance of this novel human virus.

## Methods

### DNA preparation and PCR-based genome walking

DNA was prepared from uncultured PBMCs available from person 1863LE identified in the original PTLV surveillance study in Cameroon reported in detail elsewhere [6]. DNA integrity was confirmed by β-actin polymerase chain reaction (PCR) as previously described [6]. All DNA preparation and PCR assays were performed in a laboratory where only human specimens are processed and tested according to recommended precautions to prevent contamination. To obtain the full-length genomic sequence of HTLV-4 we first PCR-amplified small regions of each major coding region by using nested PCR and degenerate PTLV primers (Fig. 1). The *tax *(730-bp), polymerase (*pol*) (662-bp), and envelope (*env*) (319-bp) sequences were amplified by using primers and conditions provided elsewhere [6,31]. An additional short HTLV-4 sequence, 440-bp in length, that overlaps the end of *tax *and the beginning of the 3'LTR was amplified using standard PCR conditions and 45°C annealing with the external primers PGTAXF7a 5'TGATGGIWSICCIATGATTTCCGG 3', PGTAXF7b 5'TGATGGGTCTCCTATGATTTCCGG3' and PGTATA1+2R1 5'TCCTGAACYGTCYYYRCGCTTTTATAG3' and the internal primers PGTAXF8 5'TGCCCIAARIMIGGICAGCCATCTTT3' and PGTATA1+2R1.

HTLV-4(1863LE)-specific primers were then designed from sequences obtained in each of the four viral regions described above and were used in nested, long-template PCRs (Expand High Fidelity kit containing both Taq and Tgo DNA polymerases (Roche)) to fill in the gaps in the genome as depicted in Fig. 1. The external and internal primer sequences for the LTR-*pol *fragment are 1863LF2 5'CCAAGGACAAAACTAGCAGGGACT3' and 1863PR4 5'GGGGATGGTAAAGGCGAAGTAGGG3', and 1863LF3 5'CGTCCCAGCCCAGCCTCAAAACCA 3'and 1863PR5 5'GGGAATCTGGAAGAAAGCGTCCGT3', respectively. The external and internal primer sequences for the *pol*-*env *fragment are 1863PF3 5'GTCCTCTCATGGTCTCCCAGTTTCCCAG 3' and 1863ER 5'GCTGGAGTGGTAGGAGGAGATAC3', and 1863PF5 5'CACTTCCTGGGCCAAATCATACATCCAGATC3' and 1863ER3 5'GGCTGGCCTGAA GTACTGGGATGCC3', respectively. The external and internal primer sequences for the *env-tax *fragment are 1863EF1 5'CCTGCCAAAACCTGATCACCTATTC3' and 1863TR1 5'CGACAACTCGTCCATCGATGG3' and 1863EF2 5'CCCTGTATCTCTTCCCACACTGGGTA C3' and 1863TR2 5'GGGGAGCATAATCCACCGGAGATGG3', respectively. The remaining 3' end of the genome was obtained by using the primers 1863pXF1 5'AACTCCGCCAATACACCCAACAGG3' and 1863LR1 5'GGAGGGGTTTGAGTACAGCGGGCT3' in a single round of PCR amplification.

PCR products were purified with a Qiaquick PCR purification kit (Qiagen), and sequenced in both directions with a BigDye terminator cycle kit and automated sequencers (Applied Biosystems). Selected PCR products were also cloned into the pCR4-TOPO vector using the TOPO TA Cloning kit (Invitrogen) and recombinant plasmid DNA was prepared using the Qiagen plasmid purification kit prior to automated sequencing.

### Sequence analysis

Percent nucleotide divergence was calculated using the GAP program in the Genetic Computer Group's (GCG) Wisconsin package [57]. Examination of functional genetic motifs involved in viral expression, regulation, and HTLV-induced oncogenesis was done by detailed comparison of the HTLV-4 genome with full-length PTLV sequences [26-29,31,44]. Identification of potential transcription factor binding sites in the HTLV-4 genome was performed using the program TESS (Transcription Element Search System) [58]. Secondary structure of the LTR RNA was determined using the program RNAstructure v4.2 program [59]. Comparison of full-length PTLV genomes available at GenBank and determination of genetic recombination was done using HTLV-4(1863LE) as the query sequence and the F84 (maximum likelihood) model and a transition/transversion ratio of 2.28 implemented in the program SimPlot [60]. Prediction of splice acceptor (sa) and splice donor (sd) sites was done using an artificial neural network implemented in the NetGene2 program [61] and with the Spliceview program [62].

Nucleotide substitution saturation was evaluated using pair-wise transition and transversion versus divergence plots using the DAMBE program [63]. Unequal nucleotide composition was measured by using the TREE-PUZZLE program [64]. Phylogenetic trees were inferred with the parameters estimated from the Clustal W [65] sequence alignments of each gene and the full-length genome after removing indels by using Modeltest v3.7 [66] and Neighbor-Joining (NJ) methods in the MEGA v4.0 [67] program and maximum-likelihood (ML) analysis in PAUP* [68], TREE-PUZZLE [64], and PhyML [69]. The reliability of the inferred tree topology was tested with 100 (PAUP*) to 1000 bootstrap replicates (NJ and PhyML) or 100,000 puzzling steps (TREE-PUZZLE). Trees were viewed and edited using FigTree v1.1.2 [70].

### PTLV evolutionary rates and divergence times

In order to estimate a reliable divergence time for the cenancestor (most recent common ancestor) of the HTLV-4(1863LE) lineage, we generated separate alignments of *gag*, *pol*, *env*, and *tax *genes from all full-length PTLV genomes available at GenBank by using Clustal W. Sequence gaps and 3^rd ^codon positions were removed, and minor adjustments in the alignment were made manually. The best fitting evolutionary model for the aligned sequences was determined using a hierarchical likelihood ratio test as described elsewhere [68]. A variant of the GTR model, allowing four different substitution rate categories (r_A↔C _= r_A↔T _= r_G↔T _= 1, r_A↔G _= 9.35, r_C↔G _= 0.67, r_C↔T _= 5.79), with gamma-distributed rate heterogeneity (a = 0.694) and an estimated proportion of invariable sites (0.185), was determined to best fit the data.

The molecular clock hypothesis, or constant rate of evolution, for the PTLV tree was tested with the likelihood ratio test [71]. Likelihoods were calculated using the best fitting nucleotide substitution model either with or without the enforcement of the global clock constraint with the program PAML [72]. The PTLV evolutionary rate assuming the global molecular clock model was estimated by using the divergence time of 40,000 – 60,000 years ago (ya) for the Melanesian HTLV-1 lineage (HTLV-1mel) and 12,000–30,000 ya for the most recent common ancestor of HTLV-2a/HTLV-2b native American strains according to the formula: evolutionary rate (*r*) = branch length (*bl*)/divergence time (*t*) [23]. Such divergence dates were based on well-established genetic and archaeological evidence suggesting that ancestors of indigenous Melanesians and Australians migrated from Southeast Asia or the introduction of ancestral indigenous Indians into North America via the Bering Straight during those times [3,4,32]. The evolutionary rate was also estimated by employing a Bayesian Markov Chain Monte Carlo (MCMC) molecular clock method, allowing for either a strict or a relaxed molecular clock [51], implemented in the BEAST software package [73]. For each analysis, we used the calibration dates discussed above as a strong prior for the time of the most recent common ancestor (tMRCA) of the HTLV-1Mel/HTLV-1a,b and HTLV-2a,b lineages, respectively. In practice, the upper and lower divergence times estimated from anthropological data were used to define the interval of a strong uniform prior distribution from which the MCMC sampler would sample possible divergence times for the corresponding node in the tree. For each model, the Bayesian calculation consisted of three independent 100,000,000 generations MCMC with sampling every 1,000^th ^generation. Convergence of the MCMC was assessed by calculating the effective sampling size (ESS) of the runs using the program Tracer [74]. All parameter estimates showed significant ESSs (>150). The tree with the maximum product of the posterior clade probabilities (maximum clade credibility tree) was chosen from the posterior distribution of 5,000 sampled trees (after burning in the first 5001 sampled trees) with the program TreeAnnotator version 1.4.6 included in the BEAST software package [73]. Both the constant coalescent and Yule Process were used as tree priors and gave identical results.

### DNA transfection

Approximately 1 million 293 cells were seeded on a 100 mm dish and incubated for 24 h at 37°C. Cells were then transfected with a c-Myb expression vector using Lipofectamine-PLUS (Invitrogen). Cells were lysed 48 hours using 1 × passive lysis buffer (Promega). Whole cell extract was stored at -80°C.

### Electrophoretic mobility shift assay (EMSA)

The double-stranded oligonucleotide probe representing the c-Myb binding site within the HTLV-4 LTR (sense, 5'-TCGAGAAAGGTCAACTGTCTCACACAAAC-3'; antisense, 3'-TCGAGTTTGTGTGAGACAGTTGACCTTTC-5') was end-labeled with [α-^32^P]dCTP using Klenow enzyme (Invitrogen). The DNA-binding reaction was incubated for 1 h at room temperature using 5 ng of labeled probe and binding buffer (10 mM Tris [pH 7.9], 50 mM NaCl, 1 mM EDTA 10 mM dithiothreitol, 0.5% non-fat dry milk, 5% glycerol) supplemented with 2 ug of sheared salmon sperm DNA, 1 ug poly-dI-dC (Sigma St. Louis, MO), and 5 ug 293 cell extract in a final volume of 15 ul. The supershift was performed by adding 1 ug of anti-c-Myb monoclonal antibody (Upstate Biotechnology, Charlottesville, VA) or non-specific PC10 monoclonal antibody (Santa Cruz Biotechnology, Santa Cruz, CA) to the binding reaction for 1 h at room temperature. Unlabeled double-stranded (sense, 5'-TCGAGAAAGGTCGTATGTCTCACACAAAC-3'; antisense, 3'-TCGAGTTTGTGTGAGACATACGACCTTTC-5') non-specific oligonucleotide contained mutations at three positions (underlined) within the predicted c-Myb binding site. Specific and non-specific competitors were added in a 100-fold excess over labeled probe. DNA-protein complexes were resolved on a 4% non-denaturing polyacrylamide gel in 0.5× Tris-borate-EDTA at 150 V for 2.5 h.

### Nucleotide sequence accession numbers

The complete HTLV-4(1863LE) proviral sequence has been deposited in GenBank with accession number EF488483. GenBank accession numbers for the complete PTLV genomes used in this paper are [HTLV-1(ATK) = J02029], [HTLV-1(ATL-YS) = U19949], [HTLV-1(Mel5) = L02534], [HTLV-1 (Boi) = L36905], [STLV-1(TE4) = Z46900], [STLV-1(Tan90) = AF074966], [HTLV-2(MoT) = M10060], [HTLV-2(Kay96) = AF356584], [HTLV-2(Gab) = Y13051], [HTLV-2(SP-WV) = AF139382], [HTLV-2(G2) = L11456], [HTLV-2(G12) = L11456], [HTLV-2(Efe) = Y14365], [STLV-2(Pan-p) = U90557], [STLV-2(pp1664) = Y14570], [HTLV-3(2026ND) = DQ093792], [HTLV-3(Pyl43) = DQ462191], [STLV-3(CTO604) = NC_003323], [STLV-3(Ph969) = Y07616], [STLV-3(TGE2117) = AY217650], [STLV-3(NG409) = AY222339], [STLV-3(Ppaf3) = AF517775], [STLV-5(MarB43) = AY590142].

## Competing interests

Some authors (WMS, NDW, DSB, TMF, WH) have applied for a patent for the discovery of HTLV-4.

## Authors' contributions

WMS conceived, designed and coordinated the study, analyzed, acquired and interpreted the data, and wrote the manuscript. MS, RRG, and AK helped design the study, performed detailed phylogenetic analysis of the sequences, and helped write the manuscript. SHQ and HJ together obtained the full-length genome of HTLV-4, analyzed the sequences, and participated in writing the manuscript. SJM and KNP helped characterize the LTR regulatory elements and participated in writing the manuscript. NDW, DSB, TMF, and WH helped design the study, assisted in analysis of the data, and participated in writing the manuscript. All authors read and approved the final manuscript.

## Supplementary Material

Additional file 1 Supplementary figures. Figure S1. Pair-wise transition (s; blue line) and transversion (v, green line) *versus *divergence plots in different HTLV-4 (1863LE) genes using 1st + 2nd or 3rd codon positions (cdp). Genetic distances were calculated with the Tamura and Nei 1993 (TN93) model and plotted against the estimated number of transitions and transversions for each pair-wise comparison using the DAMBE program. Figure S2. Evolutionary relationship of major genes and the entire genome of HTLV-4(1863LE) to other PTLVs by using either Neighbor-Joining (NJ; a-f) or maximum likelihood (ML, g-j) methods. The percentage of replicate trees in which the associated taxa clustered together in the bootstrap test (100–1000 replicates) is shown at the branch nodes. Branch lengths are drawn to scale and only bootstrap values greater than 70% are shown. Branches leading to PTLV-1, HTLV-2, and PTLV-3 sequences are drawn in red, blue, and green, respectively. The branches leading to HTLV-4(1863LE), STLV-2, and to the divergent STLV-5(MarB43) strain are drawn in magenta, purple, and yellow, respectively. Click here for file
