# Supplementary material for: Ancient, independent evolution and distinct molecular features of the novel human T-lymphotropic virus type 4
Source: Retrovirology. 2009 Feb 2;6:9. doi: 10.1186/1742-4690-6-9 (PMC2647524; doi:10.1186/1742-4690-6-9)
Supplement: Additional file 1 — Supplementary figures. Figure S1. Pair-wise transition (s; blue line) and transversion (v, green line) versus divergence plots in different HTLV-4 (1863LE) genes using 1st + 2nd or 3rd codon positions (cdp). Genetic distances were calculated with the Tamura and Nei 1993 (TN93) model and plotted against the estimated number of transitions and transversions for each pair-wise comparison using the DAMBE program. Figure S2. Evolutionary relationship of major genes and the entire genome of HTLV-4(1863LE) to other PTLVs by using either Neighbor-Joining (NJ; a-f) or maximum likelihood (ML, g-j) methods. The percentage of replicate trees in which the associated taxa clustered together in the bootstrap test (100–1000 replicates) is shown at the branch nodes. Branch lengths are drawn to scale and only bootstrap values greater than 70% are shown. Branches leading to PTLV-1, HTLV-2, and PTLV-3 sequences are drawn in red, blue, and green, respectively. The branches leading to HTLV-4(1863LE), STLV-2, and to the divergent STLV-5(MarB43) strain are drawn in magenta, purple, and yellow, respectively. [file 1742-4690-6-9-S1.ppt]

## Slide 1
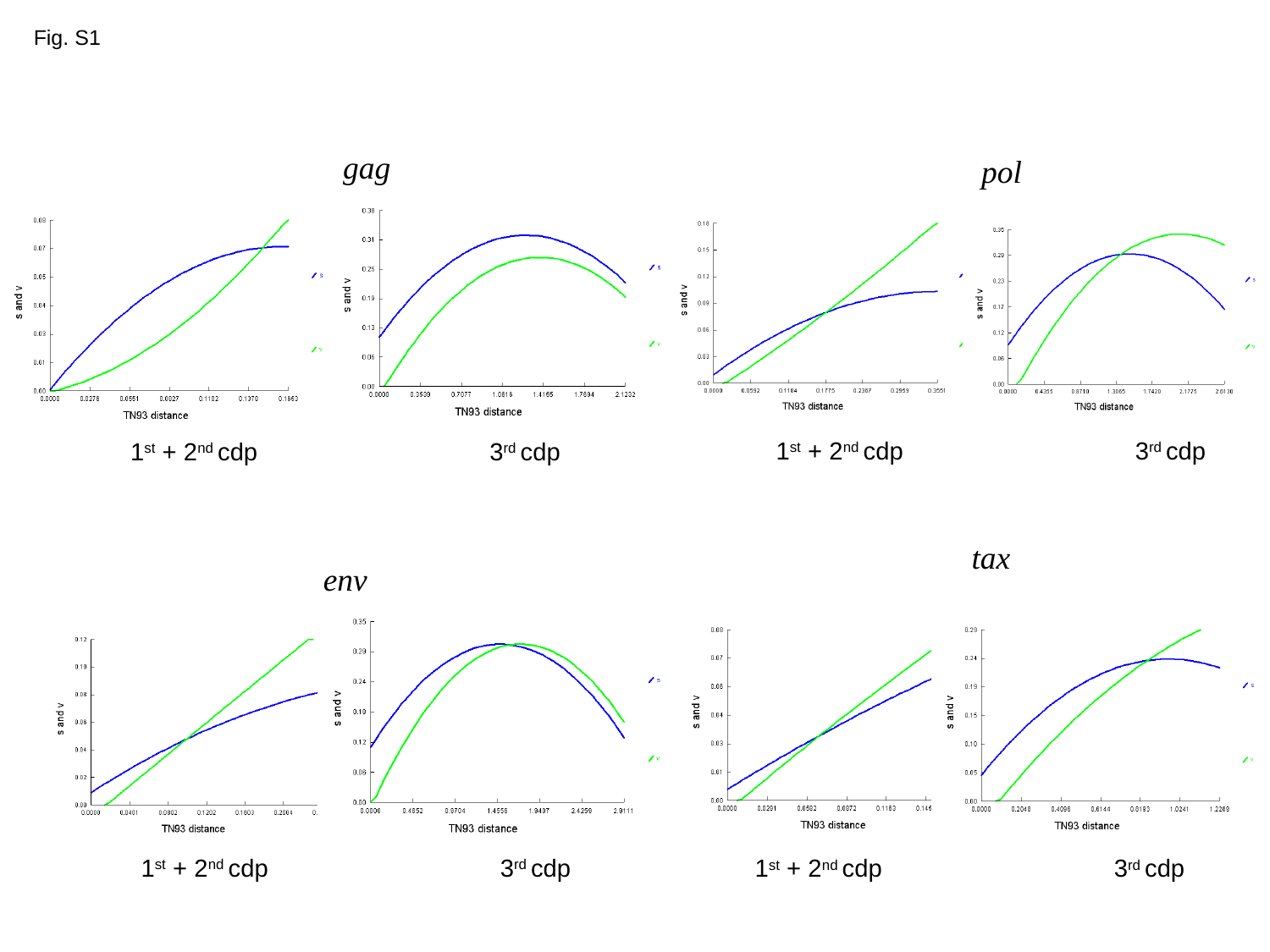

Fig. S1
gag
pol
1st + 2nd cdp
3rd cdp
1st + 2nd cdp
3rd cdp
tax
# env
1st + 2nd cdp
3rd cdp
1st + 2nd cdp
3rd cdp

## Slide 2
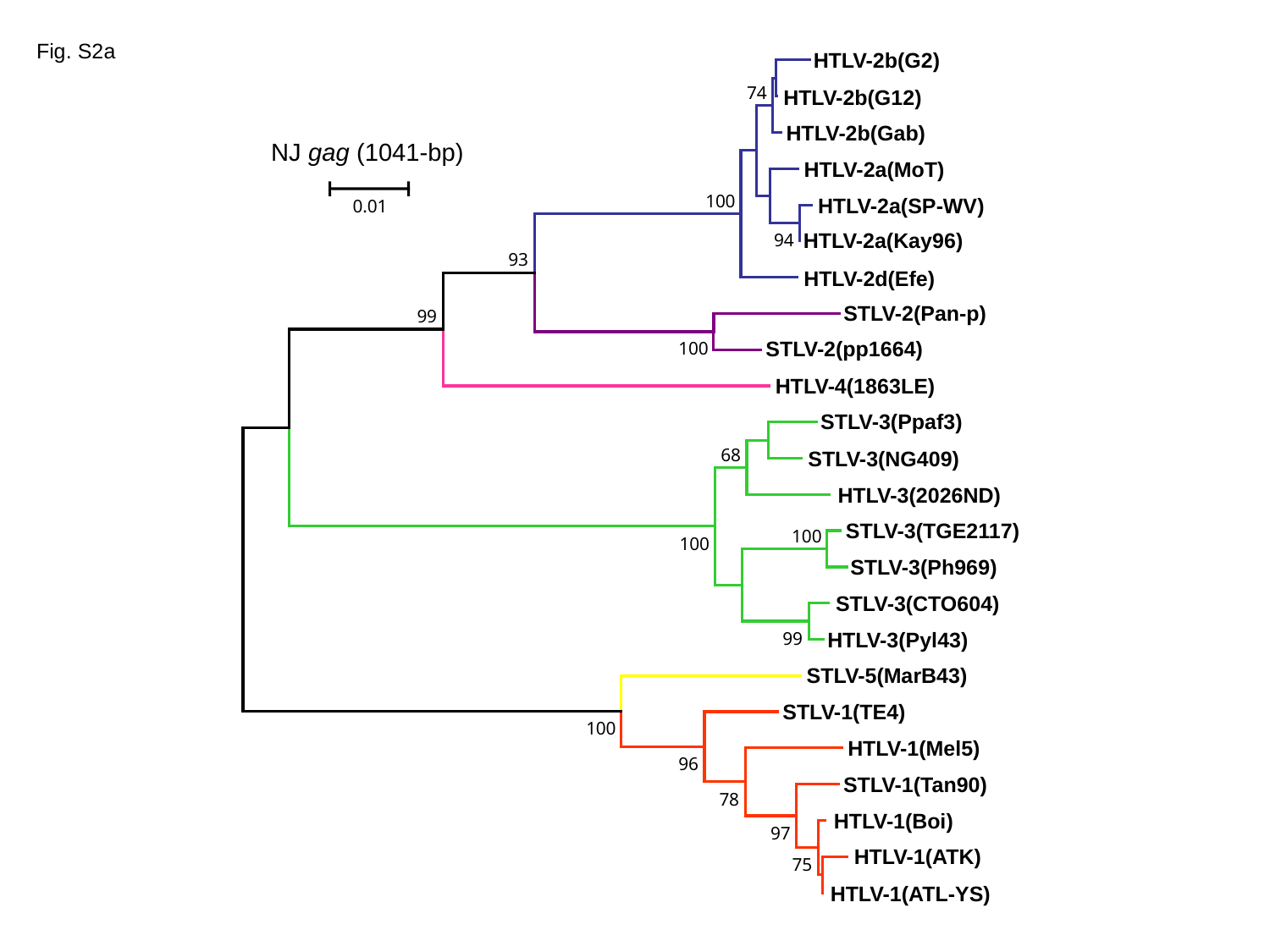

Fig. S2a
HTLV-2b(G2)
74
HTLV-2b(G12)
HTLV-2b(Gab)
NJ gag (1041-bp)
HTLV-2a(MoT)
100
HTLV-2a(SP-WV)
0.01
HTLV-2a(Kay96)
94
93
HTLV-2d(Efe)
STLV-2(Pan-p)
99
STLV-2(pp1664)
100
HTLV-4(1863LE)
STLV-3(Ppaf3)
68
STLV-3(NG409)
HTLV-3(2026ND)
STLV-3(TGE2117)
100
100
STLV-3(Ph969)
STLV-3(CTO604)
HTLV-3(Pyl43)
99
STLV-5(MarB43)
STLV-1(TE4)
100
HTLV-1(Mel5)
96
STLV-1(Tan90)
78
HTLV-1(Boi)
97
HTLV-1(ATK)
75
HTLV-1(ATL-YS)

## Slide 3
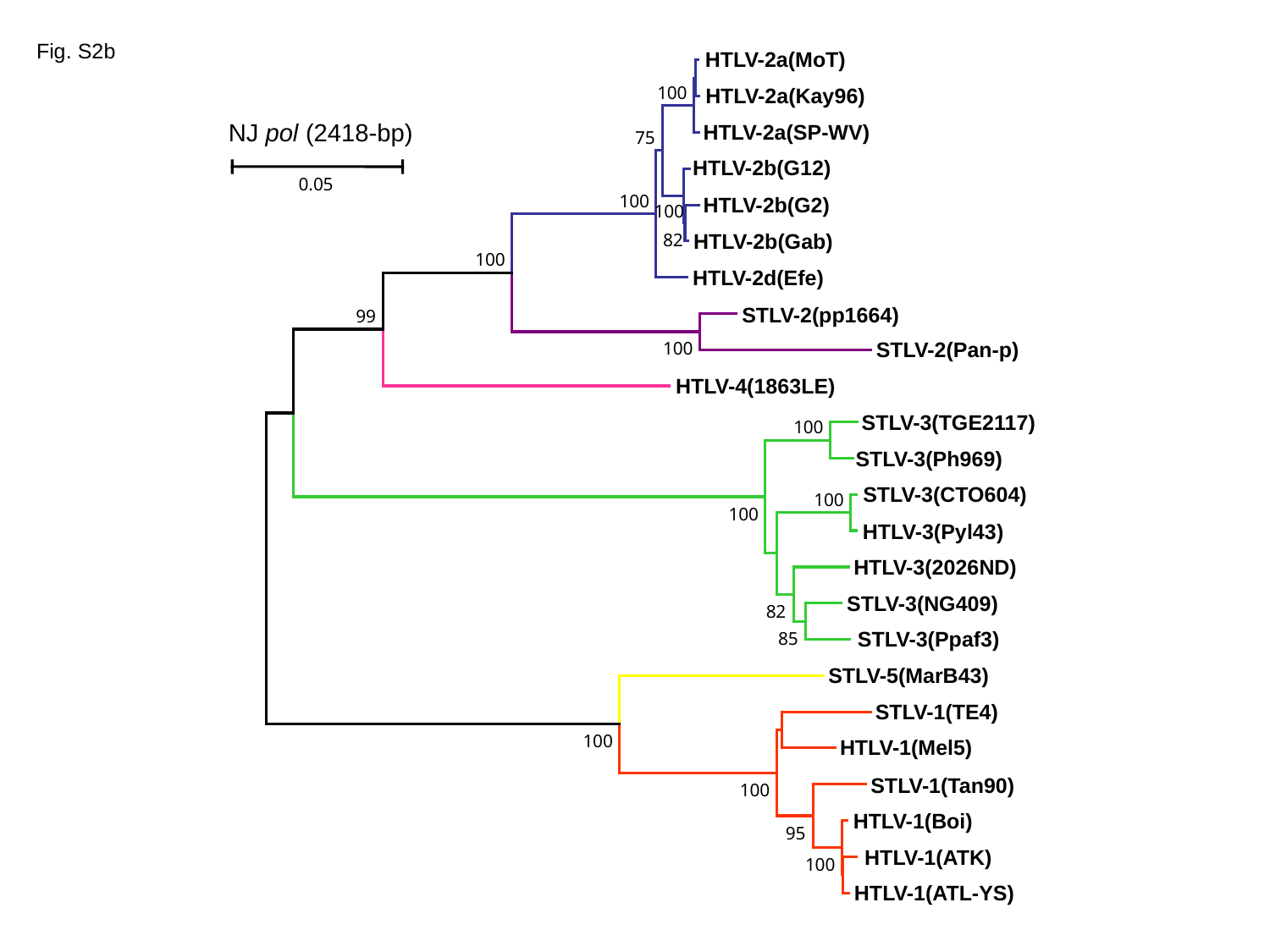

Fig. S2b
HTLV-2a(MoT)
100
HTLV-2a(Kay96)
NJ pol (2418-bp)
HTLV-2a(SP-WV)
75
HTLV-2b(G12)
0.05
100
HTLV-2b(G2)
100
HTLV-2b(Gab)
82
100
HTLV-2d(Efe)
STLV-2(pp1664)
99
STLV-2(Pan-p)
100
HTLV-4(1863LE)
STLV-3(TGE2117)
100
STLV-3(Ph969)
STLV-3(CTO604)
100
100
HTLV-3(Pyl43)
HTLV-3(2026ND)
STLV-3(NG409)
82
STLV-3(Ppaf3)
85
STLV-5(MarB43)
STLV-1(TE4)
100
HTLV-1(Mel5)
STLV-1(Tan90)
100
HTLV-1(Boi)
95
HTLV-1(ATK)
100
HTLV-1(ATL-YS)

## Slide 4
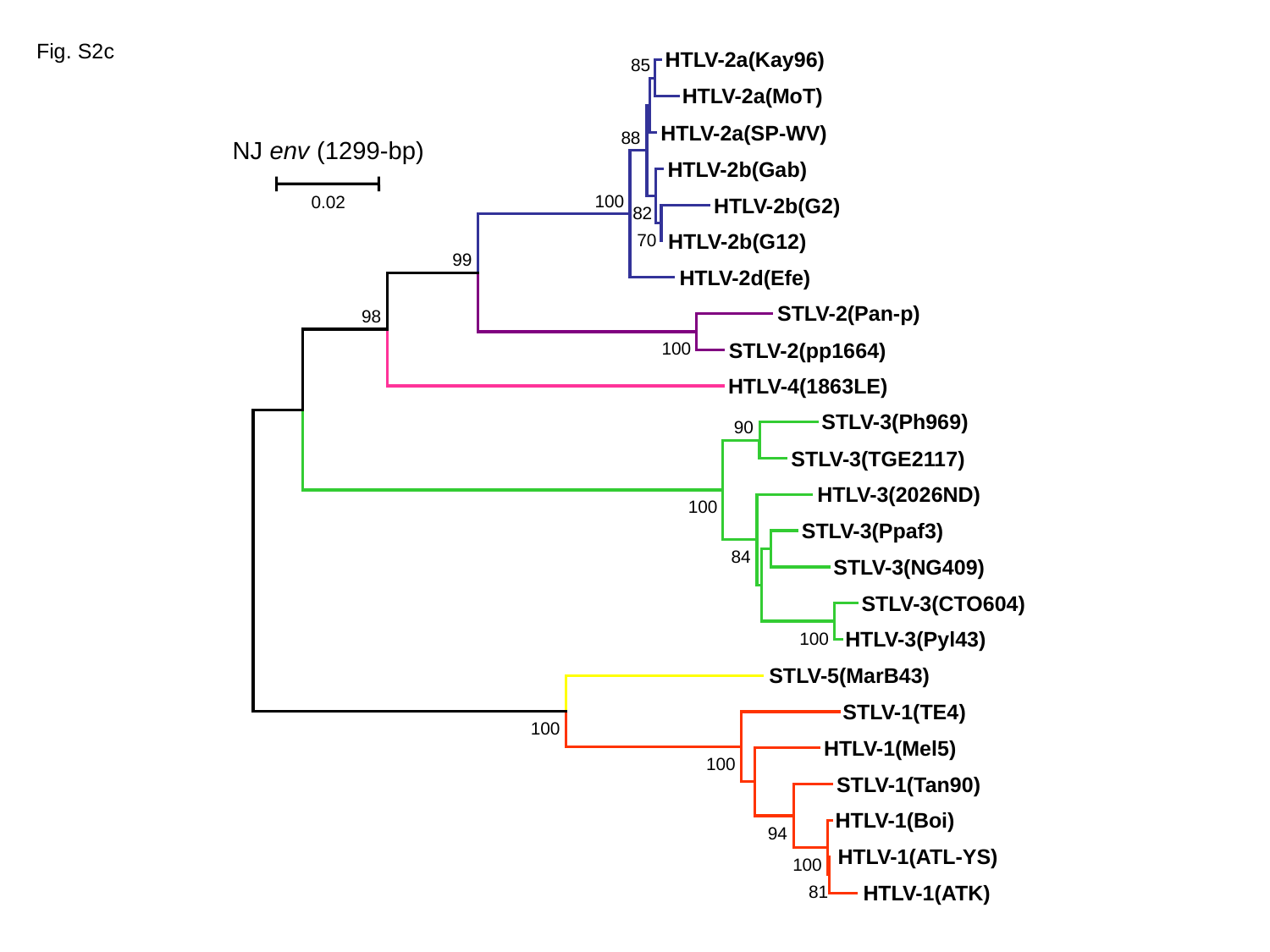

Fig. S2c
HTLV-2a(Kay96)
85
HTLV-2a(MoT)
HTLV-2a(SP-WV)
88
NJ env (1299-bp)
HTLV-2b(Gab)
100
0.02
HTLV-2b(G2)
82
HTLV-2b(G12)
70
99
HTLV-2d(Efe)
STLV-2(Pan-p)
98
STLV-2(pp1664)
100
HTLV-4(1863LE)
STLV-3(Ph969)
90
STLV-3(TGE2117)
HTLV-3(2026ND)
100
STLV-3(Ppaf3)
84
STLV-3(NG409)
STLV-3(CTO604)
HTLV-3(Pyl43)
100
STLV-5(MarB43)
STLV-1(TE4)
100
HTLV-1(Mel5)
100
STLV-1(Tan90)
HTLV-1(Boi)
94
HTLV-1(ATL-YS)
100
HTLV-1(ATK)
81

## Slide 5
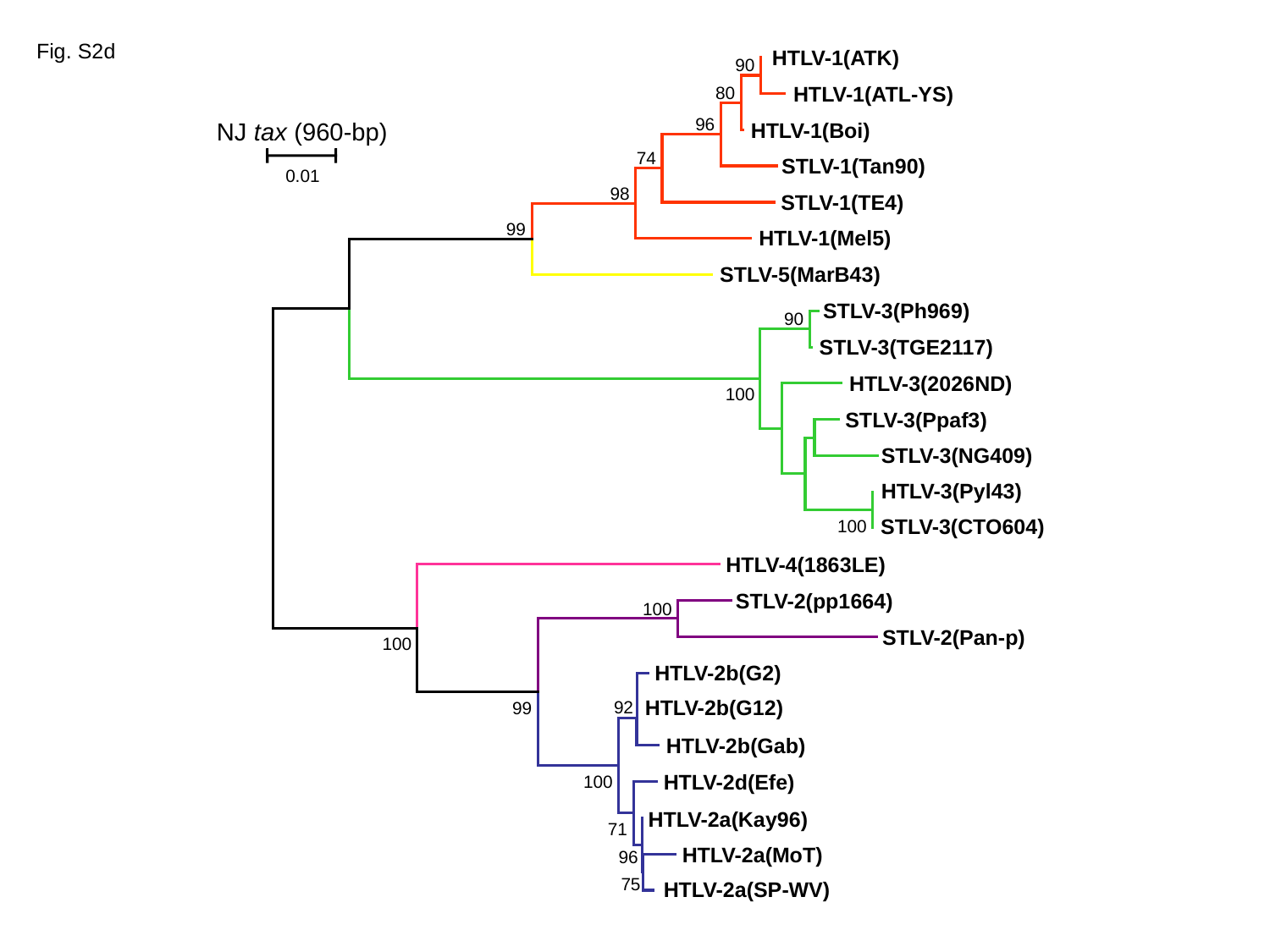

Fig. S2d
HTLV-1(ATK)
90
HTLV-1(ATL-YS)
80
NJ tax (960-bp)
96
HTLV-1(Boi)
74
STLV-1(Tan90)
0.01
98
STLV-1(TE4)
99
HTLV-1(Mel5)
STLV-5(MarB43)
STLV-3(Ph969)
90
STLV-3(TGE2117)
HTLV-3(2026ND)
100
STLV-3(Ppaf3)
STLV-3(NG409)
HTLV-3(Pyl43)
STLV-3(CTO604)
100
HTLV-4(1863LE)
STLV-2(pp1664)
100
STLV-2(Pan-p)
100
HTLV-2b(G2)
HTLV-2b(G12)
92
99
HTLV-2b(Gab)
HTLV-2d(Efe)
100
HTLV-2a(Kay96)
71
HTLV-2a(MoT)
96
75
HTLV-2a(SP-WV)

## Slide 6
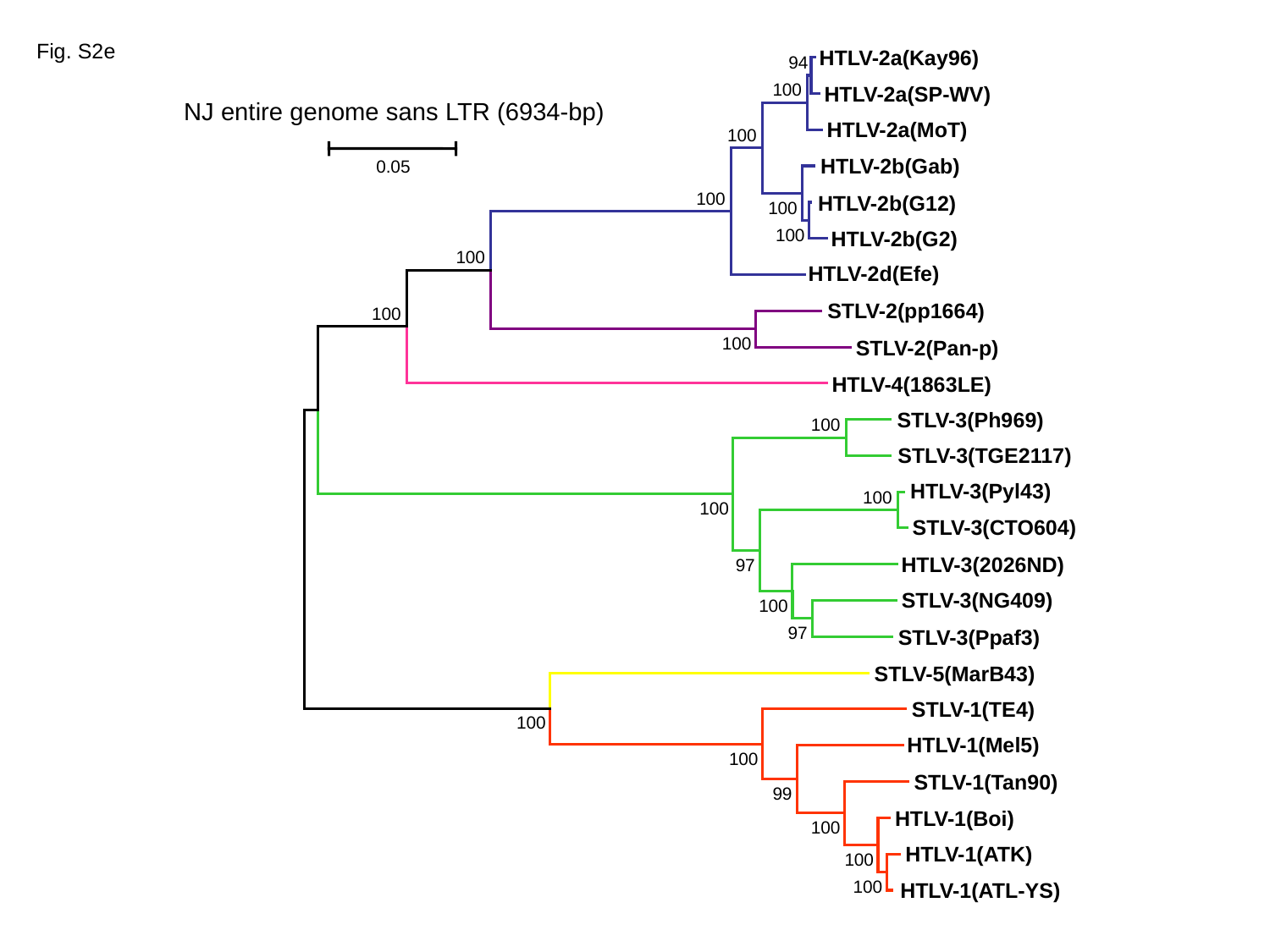

Fig. S2e
HTLV-2a(Kay96)
94
100
HTLV-2a(SP-WV)
NJ entire genome sans LTR (6934-bp)
HTLV-2a(MoT)
100
HTLV-2b(Gab)
0.05
100
HTLV-2b(G12)
100
100
HTLV-2b(G2)
100
HTLV-2d(Efe)
STLV-2(pp1664)
100
100
STLV-2(Pan-p)
HTLV-4(1863LE)
STLV-3(Ph969)
100
STLV-3(TGE2117)
HTLV-3(Pyl43)
100
100
STLV-3(CTO604)
HTLV-3(2026ND)
97
STLV-3(NG409)
100
97
STLV-3(Ppaf3)
STLV-5(MarB43)
STLV-1(TE4)
100
HTLV-1(Mel5)
100
STLV-1(Tan90)
99
HTLV-1(Boi)
100
HTLV-1(ATK)
100
100
HTLV-1(ATL-YS)

## Slide 7
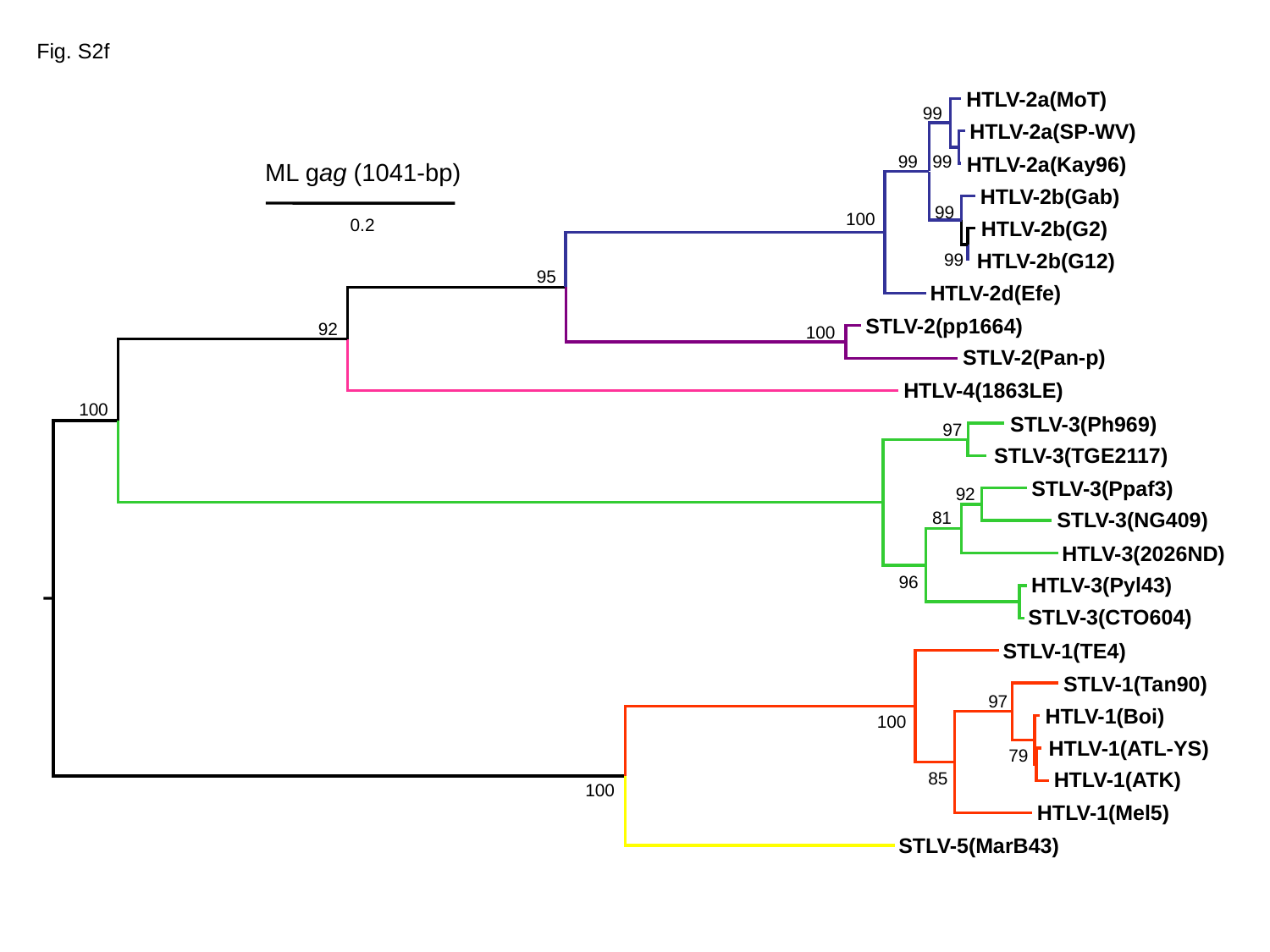

Fig. S2f
HTLV-2a(MoT)
99
HTLV-2a(SP-WV)
99
ML gag (1041-bp)
99
HTLV-2a(Kay96)
HTLV-2b(Gab)
99
0.2
100
HTLV-2b(G2)
HTLV-2b(G12)
99
95
HTLV-2d(Efe)
STLV-2(pp1664)
92
100
STLV-2(Pan-p)
HTLV-4(1863LE)
100
STLV-3(Ph969)
97
STLV-3(TGE2117)
STLV-3(Ppaf3)
92
81
STLV-3(NG409)
HTLV-3(2026ND)
96
HTLV-3(Pyl43)
STLV-3(CTO604)
STLV-1(TE4)
STLV-1(Tan90)
97
HTLV-1(Boi)
100
HTLV-1(ATL-YS)
79
HTLV-1(ATK)
85
100
HTLV-1(Mel5)
STLV-5(MarB43)

## Slide 8
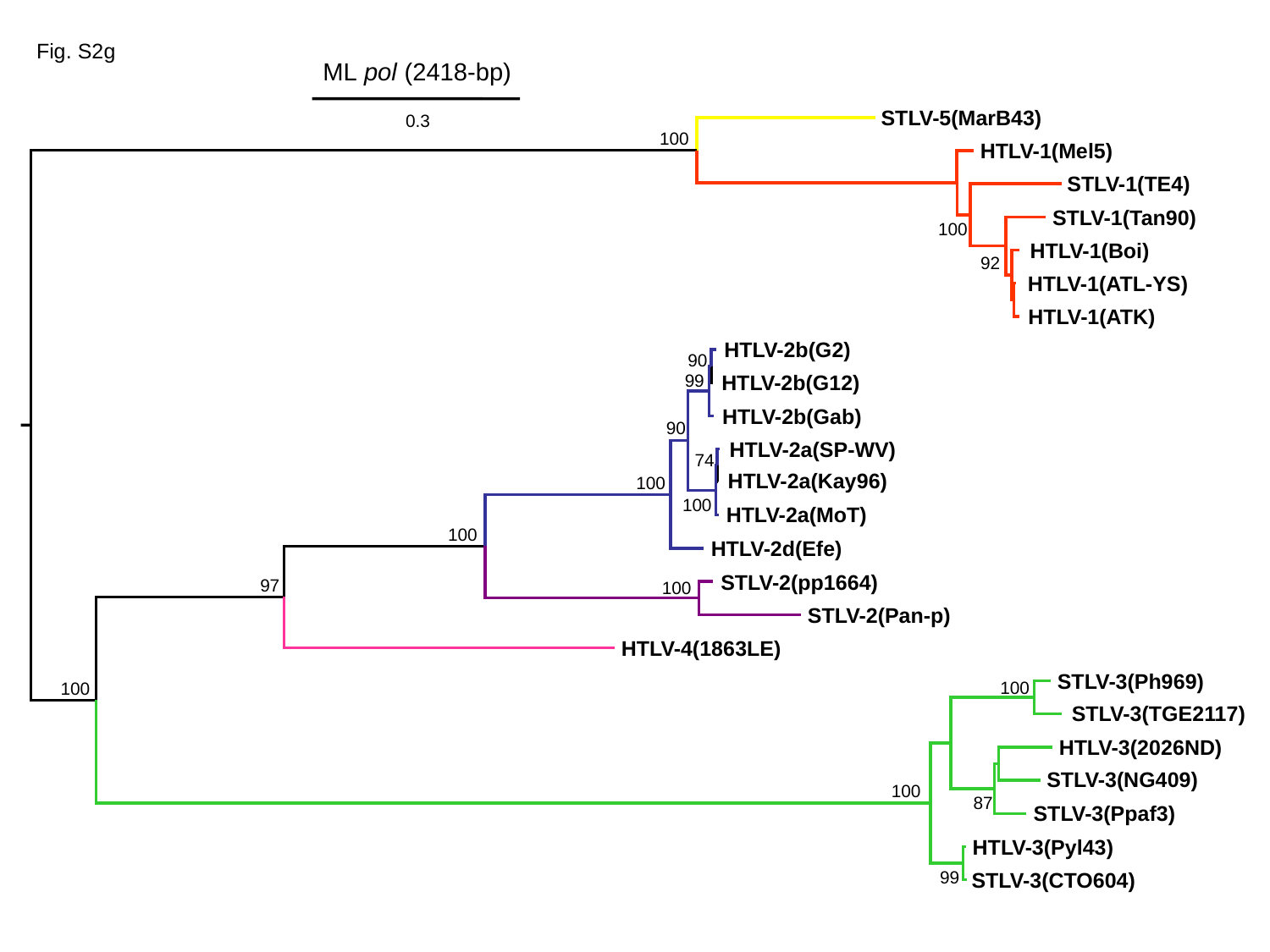

Fig. S2g
ML pol (2418-bp)
0.3
STLV-5(MarB43)
100
HTLV-1(Mel5)
STLV-1(TE4)
STLV-1(Tan90)
100
HTLV-1(Boi)
92
HTLV-1(ATL-YS)
HTLV-1(ATK)
HTLV-2b(G2)
90
99
HTLV-2b(G12)
HTLV-2b(Gab)
90
HTLV-2a(SP-WV)
74
HTLV-2a(Kay96)
100
100
HTLV-2a(MoT)
100
HTLV-2d(Efe)
STLV-2(pp1664)
97
100
STLV-2(Pan-p)
HTLV-4(1863LE)
STLV-3(Ph969)
100
100
STLV-3(TGE2117)
HTLV-3(2026ND)
STLV-3(NG409)
100
87
STLV-3(Ppaf3)
HTLV-3(Pyl43)
99
STLV-3(CTO604)

## Slide 9
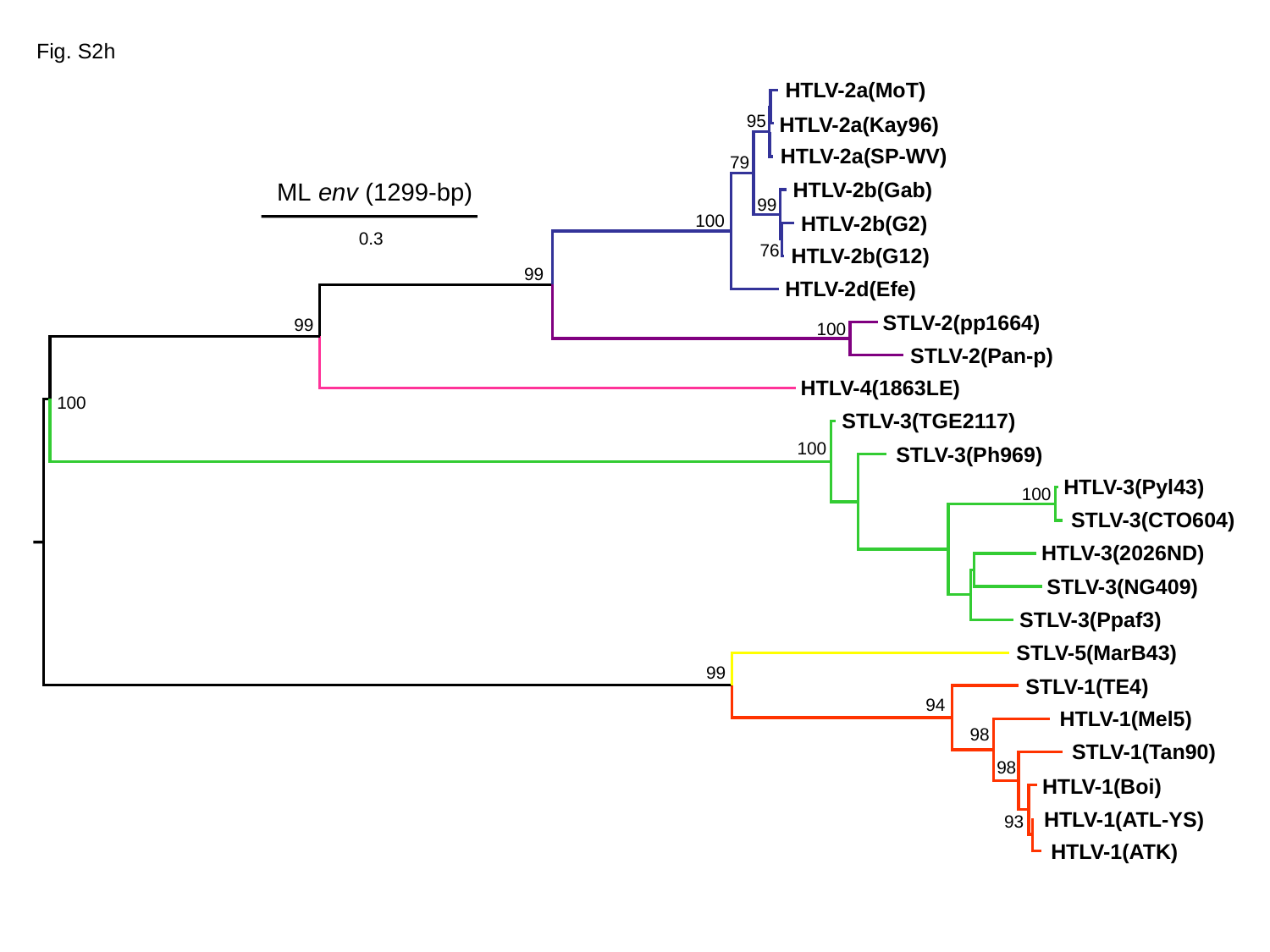

Fig. S2h
HTLV-2a(MoT)
95
HTLV-2a(Kay96)
HTLV-2a(SP-WV)
79
ML env (1299-bp)
HTLV-2b(Gab)
99
100
HTLV-2b(G2)
0.3
76
HTLV-2b(G12)
99
HTLV-2d(Efe)
STLV-2(pp1664)
99
100
STLV-2(Pan-p)
HTLV-4(1863LE)
100
STLV-3(TGE2117)
100
STLV-3(Ph969)
HTLV-3(Pyl43)
100
STLV-3(CTO604)
HTLV-3(2026ND)
STLV-3(NG409)
STLV-3(Ppaf3)
STLV-5(MarB43)
99
STLV-1(TE4)
94
HTLV-1(Mel5)
98
STLV-1(Tan90)
98
HTLV-1(Boi)
HTLV-1(ATL-YS)
93
HTLV-1(ATK)

## Slide 10
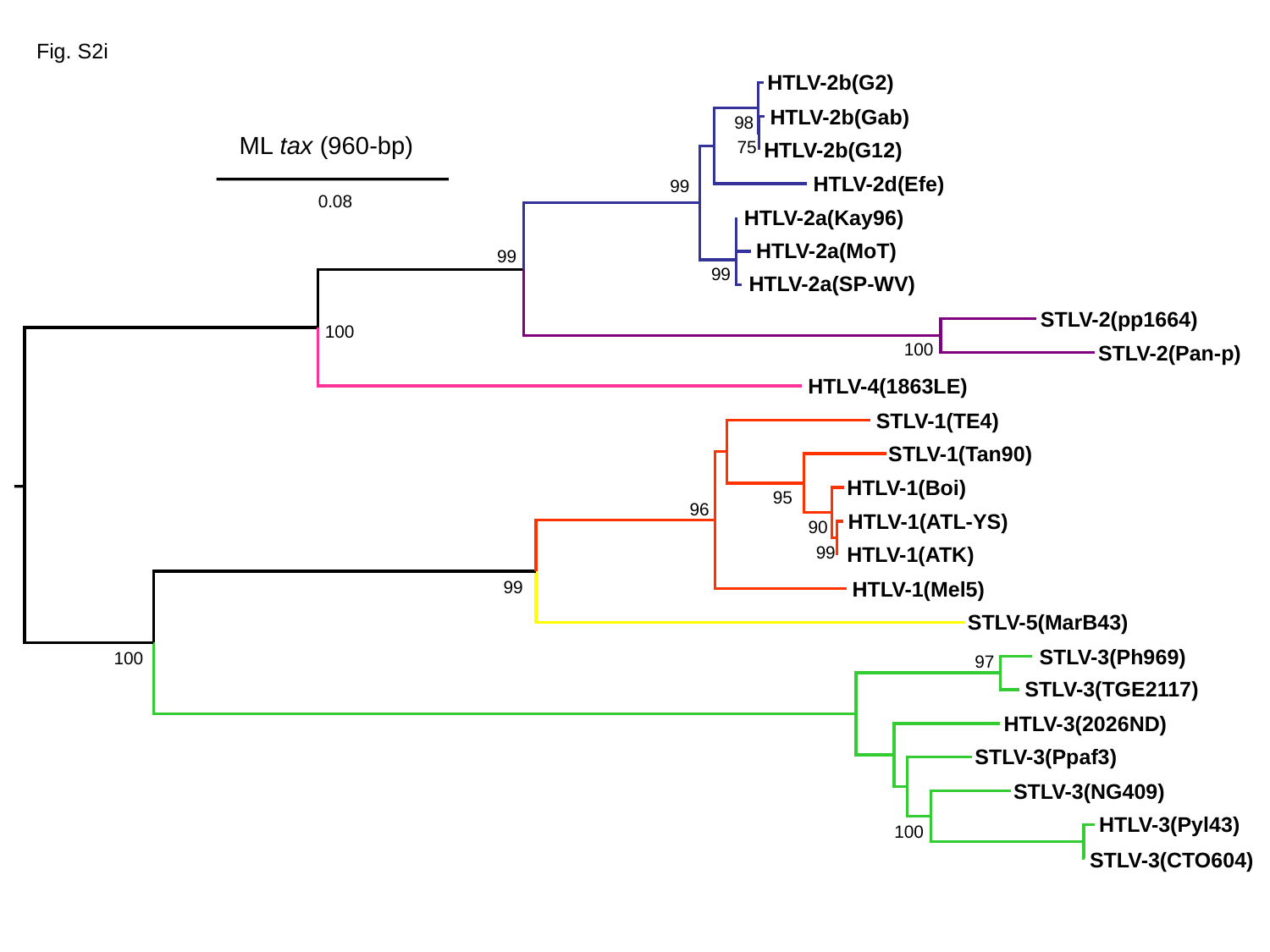

Fig. S2i
HTLV-2b(G2)
HTLV-2b(Gab)
98
ML tax (960-bp)
75
HTLV-2b(G12)
HTLV-2d(Efe)
99
0.08
HTLV-2a(Kay96)
HTLV-2a(MoT)
99
99
HTLV-2a(SP-WV)
STLV-2(pp1664)
100
100
STLV-2(Pan-p)
HTLV-4(1863LE)
STLV-1(TE4)
STLV-1(Tan90)
HTLV-1(Boi)
95
96
HTLV-1(ATL-YS)
90
99
HTLV-1(ATK)
99
HTLV-1(Mel5)
STLV-5(MarB43)
STLV-3(Ph969)
100
97
STLV-3(TGE2117)
HTLV-3(2026ND)
STLV-3(Ppaf3)
STLV-3(NG409)
HTLV-3(Pyl43)
100
STLV-3(CTO604)

## Slide 11
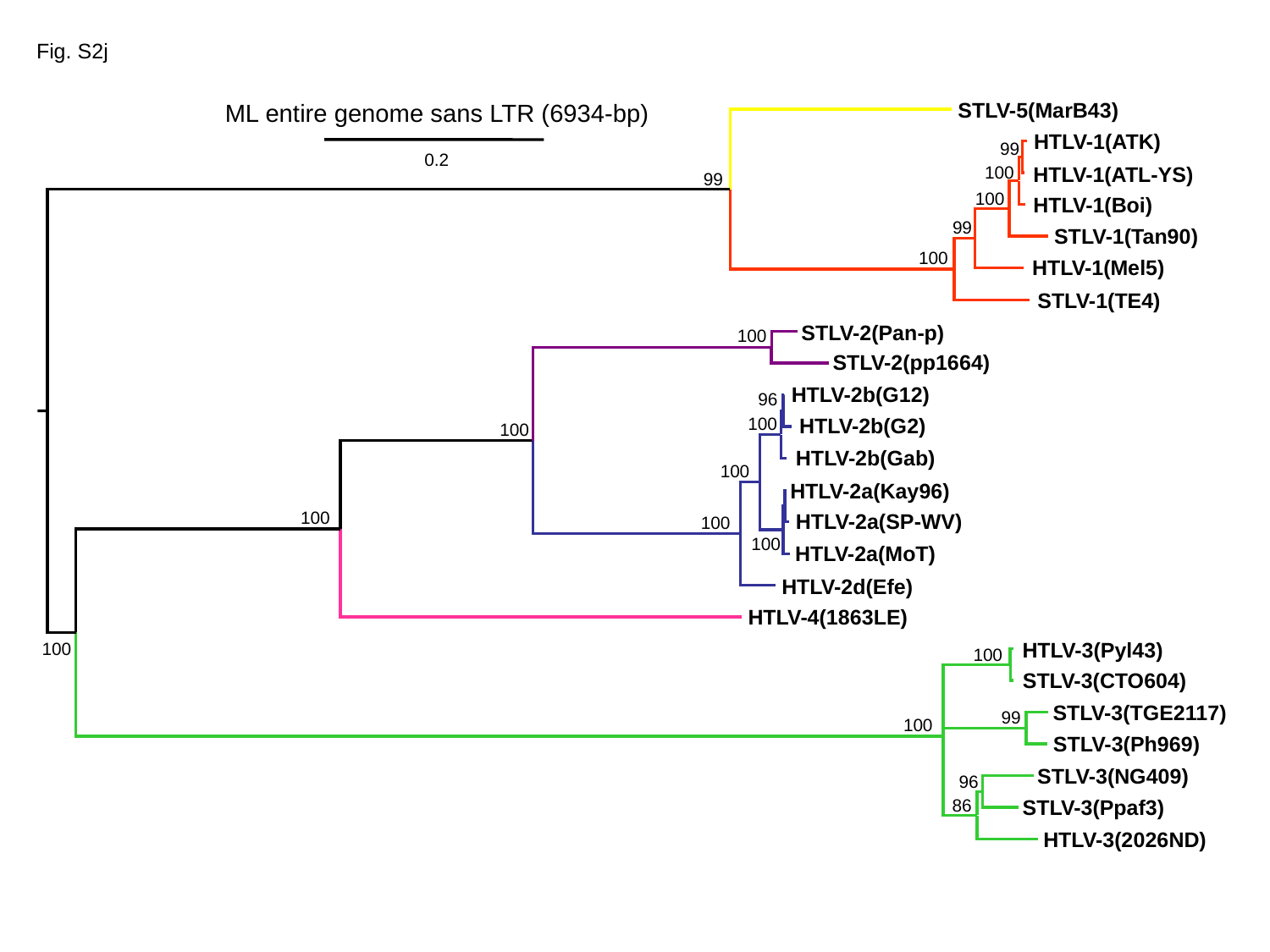

Fig. S2j
ML entire genome sans LTR (6934-bp)
STLV-5(MarB43)
HTLV-1(ATK)
99
0.2
100
HTLV-1(ATL-YS)
99
100
HTLV-1(Boi)
99
STLV-1(Tan90)
100
HTLV-1(Mel5)
STLV-1(TE4)
STLV-2(Pan-p)
100
STLV-2(pp1664)
HTLV-2b(G12)
96
100
HTLV-2b(G2)
100
HTLV-2b(Gab)
100
HTLV-2a(Kay96)
100
HTLV-2a(SP-WV)
100
100
HTLV-2a(MoT)
HTLV-2d(Efe)
HTLV-4(1863LE)
HTLV-3(Pyl43)
100
100
STLV-3(CTO604)
STLV-3(TGE2117)
99
100
STLV-3(Ph969)
STLV-3(NG409)
96
STLV-3(Ppaf3)
86
HTLV-3(2026ND)
